# Association between lipid levels and all-cause and cause-specific mortality in critically ill patients

**DOI:** 10.1038/s41598-023-32209-z

**Published:** 2023-03-29

**Authors:** Shan Li, Wei Zhang, Hongbin Liu

**Affiliations:** 1 Department of Cardiology, The Second Medical Center, Chinese People Liberation Army General Hospital, Beijing, China; 2 National Clinical Research Center for Geriatric Disease, Beijing, China

**Keywords:** Prognostic markers, Outcomes research

## Abstract

Extremely low lipid levels are considered a sign of debilitation and illness. The association between lipid levels and the risk of mortality in critically ill patients has not been well investigated. This study was designed to evaluate the association between lipid levels and all-cause and cause-specific mortality in critically ill patients using a large collaborative research database known as the eICU database. In total, 27,316 individuals with low-density lipoprotein cholesterol (LDL-C), high-density lipoprotein cholesterol (HDL-C), total cholesterol (TC) and triglyceride (TG) measurements were analyzed. A J-shaped association was observed between LDL-C, HDL-C, and TC levels and all-cause and noncardiovascular mortality, with low concentrations associated with higher risk. LDL-C, HDL-C and TC levels in the first quintile were associated with higher all-cause and noncardiovascular mortality but not with cardiovascular mortality compared to the reference quintile. There was a marked synergistic effect between low LDL-C combined with low HDL-C on the risk of mortality. Individuals with LDL-C ≤ 96 mg/dL and HDL-C ≤ 27 mg/dL had an increased risk of all-cause mortality (OR 1.52, 95% CI: 1.26–1.82), cardiovascular mortality (OR 1.07, 95% CI: 1.37–1.76) and noncardiovascular mortality (OR 1.82, 95% CI: 1.37–2.43). The results of this observational cohort showed that low LDL-C, HDL-C and TC levels were independently associated with higher all-cause and noncardiovascular mortality in critically ill patients.

## Introduction

Dyslipidemia is a recognized risk factor for cardiovascular events in individuals with atherosclerosis cardiovascular diseases (ASCVD), and the concept of ‘lower cholesterol, better prognosis’ has been proposed to prevent adverse cardiovascular events and subsequent mortality^1–5^. With increasing evidence on the benefits of intensive lipid-lowering therapy, recent clinical guidelines have recommended lower lipid-lowering goals and a wider target population^6,7^. However, reverse epidemiology or lipid paradoxes have been discussed in various clinical settings. Some studies have reported U-shaped or J-shaped associations, negative associations or null associations^8–12^ between lipid levels and the risk of death, with results varying across populations.

Low lipid levels are considered a substitute for debilitation and illness. Although the relationship between lipids and clinical outcomes has been investigated in different populations, few studies have focused on critically ill patients, a group at greater risk of death. In addition, most studies have assessed the association between lipid levels and long-term death. A previous study revealed that low LDL-C levels are correlated with increased in-hospital mortality in patients with acute myocardial infarction, contrary to findings outside the acute setting^12^. Therefore, the pattern of association between lipids and short-term risk may differ from that of long-term risk. This study sought to determine the association between four lipid components (LDL-C, HDL-C, TC and TG) and 30-day all-cause and cause-specific mortality in critically ill patients in a large intensive care unit (ICU) database.

## Method

### Data source

The eICU Collaborative Research Database (eICU-CRD) served as the data source^13^. The database was developed by the computational physiology laboratory of the Massachusetts Institute of Technology (Cambridge, MA, USA), and version 2.0 was released in 2019. This large multicenter database incorporates more than 200,000 admission records from 335 ICU wards in 208 U.S. hospitals between 2014 and 2015, including an array of demographic characteristics, admission diagnoses, physiologic parameters, comorbidity burden, laboratory measurements and treatment information. The data are freely available after completing the specified course in research with human subjects and signing the use agreement. All the data sources are deidentified, informed consent was waived from all patients and/or their legal guardians. The study was conducted in accordance with the Declaration of Helsinki and the use of this database was approved by the institutional review board of Massachusetts Institute of Technology. The institutional review board of the Massachusetts Institute of Technology waived the requirement for written informed consent. One author (Shan Li) obtained the access and was responsible for the data extraction (certification number: 46622370). The current study is reported in accordance with the Strengthening the Reporting of Observational Studies in Epidemiology (STROBE) guidelines^14^.

### Patient selection

All individuals who had at least one complete fasting lipid panel (LDL-C, HDL-C, TC, TG) measurement in the eICU-CRD were eligible for analysis. Data from the first measurement after hospitalization were used for the main analysis, and the first measurement after ICU admission was used for sensitivity analysis. Finally, 27,316 individuals were included and analyzed.

### Exposure and outcome

The primary exposure of interest was the first measurement of fasting lipid levels after hospitalization, and LDL-C, HDL-C, TC and TG levels were categorized into quintiles to investigate the association between lipid levels and outcomes. TC, HDL-C and TG levels were measured directly. LDL-C was calculated by the Friedewald equation: LDL-C = total cholesterol- HDL-C- triglycerides/5 in mg/dL (or total cholesterol- HDL-C- triglyceride/2.2 in mmol/L) when triglyceride levels were below 400 mg/dL or otherwise measured directly.

The primary outcome was all-cause mortality, and the secondary outcomes were cardiovascular mortality and noncardiovascular mortality. ICU episode data were identified by the International Classification of Diseases, Ninth Revision codes (ICD-9). All-cause death was defined as death from any cause, cardiovascular death was defined as death from ICD-9 codes 390 to 459, and other causes of death were defined as noncardiovascular death.

### Covariate selection

Covariates included age, sex, ethnicity, body mass index (BMI), heart rate, mean blood pressure, disease severity score (Acute Physiology, Age, and Chronic Health Evaluation [APACHE] score, Glasgow Coma Score [GCS]), lab measurement (LDL-C, HDL-C, total cholesterol, triglyceride, white blood cell, hemoglobin, platelet and albumin), primary disease of admission (circulatory disease, respiratory disease, digestive disease, neurological disease, genitourinary disease and endocrine disease), pre-admission comorbidities (ASCVD, diabetes, hypertension, heart failure, chronic obstructive pulmonary disease [COPD] and renal dysfunction), medication (lipid-lowering therapy, antiplatelet drug and anticoagulant), mechanical ventilation and dialysis. The APACHE IV system is a tool to predict in-hospital mortality of ICU patients and evaluate ICU performance^15^. Lipid-lowering drugs included statins (99.0%, 9666 of 9761), ezetimibe, fibrate and niacin but not proprotein convertase subtilisin/kexin type 9 (PCSK9) inhibitors, as they were not available at the time. Antiplatelet drugs included aspirin, clopidogrel, ticagrelor, prasugrel and cilostazol. Anticoagulants included warfarin, rivaroxaban and dabigatran, and other novel oral anticoagulants were not available at the time.

### Statistical analysis

Continuous variables and categorical variables were described as the means with standard deviation (SD) or medians with interquartile range (IQR) and number with frequency (%), and baseline characteristics were compared using the Mann‒Whitney U test and Pearson’s chi-square test as appropriate. Covariates were analyzed as continuous variables where appropriate, unless otherwise stated. The data integrity of most covariates was above 95%, and missing values were indicated by dummy variables.

The association between each lipid component and all-cause and cause-specific mortality was examined on a categorical scale according to the quintiles of lipid concentrations by a multivariable logistic regression model. The reference group was the quintile associated with the lowest risk of death. The association between each lipid component and all-cause and cause-specific mortality was also analyzed on a continuous scale by spline curves based on the generalized additive model. Threshold analysis was assessed by the segmented regression model and likelihood ratio test, with one knot at inflection points. Subsequently, we classified LDL-C and HDL-C levels according to identified inflection points and created a 4-category variable to assess the synergistic effect, adjusting the predefined covariates in the model.

Stratified analysis and interaction of lipid levels with age, sex, ethnicity, lipid-lowering therapy, ASCVD, LDL-C and HDL-C levels on all-cause mortality were evaluated by incorporating a two-factor interaction term in a multivariable logistic regression model to examine if the association was modified by stratified covariates.

Several sensitivity analyses were performed to assess the robustness of the main results by treating lipid levels as continuous or categorical variables. First, we excluded deaths within the first 24 h of ICU entry to determine whether the association was attributable to reverse causation due to severe illness. Second, we conducted a complete-case analysis that included only complete data of all covariates to check whether the missing data modified the main results. Third, we used the first measurement of lipid levels after ICU admission instead of the first measurement after hospital admission to assess whether the measurement at different times changed the results. Fourth, we constructed the Kaplan‒Meier survival curve with the length of ICU stay as the underlying timeline and used the log-rank test for comparison to investigate whether the main results were consistent with a different statistical method.

Statistical analyses were conducted by R version 3.6.1 software (R Project for Statistical Computing; http://www.r-project.org) and EmpowerStats (http://www.empowerstats.com, X&Y Solutions, Inc., Boston, MA). Statistical significance was set as a 2-sided P value < 0.05.

## Results

### Baseline characteristics

Among the 27,316 individuals included in the study, a total of 1704 (6.2%) all-cause deaths, 960 (3.5%) cardiovascular deaths and 744 (2.7%) noncardiovascular deaths were recorded within 30 days of ICU entry. Compared with individuals with high LDL-C levels, those with low LDL-C levels were older; more likely to be male; and had a higher APACHE score, lower TC, higher heart rate, lower blood pressure, lower prevalence of circulatory disease, higher prevalence of respiratory disease and digestive disease, and more mechanical ventilation and dialysis. Individuals with low TC levels also had similar characteristics. Individuals with low HDL-C levels were younger; more likely to be male; and had a higher APACHE score, lower TC, higher TG, higher heart rate, lower blood pressure, higher prevalence of digestive disease and more mechanical ventilation treatment. For TG, there was no significant difference in heart rate, the prevalence of circulatory disease or ventilation and dialysis. Among those at the lowest LDL-C, HDL-C, TC and TG quintiles, all-cause deaths were reported to be 10.7%, 9.3%, 10.7% and 6.8%, respectively. Cardiovascular deaths were 4.9%, 4.0%, 4.6% and 4.0%, and noncardiovascular deaths were 5.8%, 5.4%, 6.1% and 2.8%, respectively. The baseline characteristics of individual lipid components by quintiles are shown in Table 1 and eTable 1–3.

**Table 1:** Baseline characteristics of individuals according to quintile of LDL-C levels.

Baseline LDL-C	1st Quintile ≤ 50 mg/dL	2nd Quintile 51–69 mg/dL	3rd Quintile 70–88 mg/dL	4th Quintile 89–114 mg/dL	5th Quintile ≥ 115 mg/dL
N(%)	5258 (19.2)	5539 (20.3)	5447 (19.9)	5568 (20.4)	5504 (20.1)
Age, years	68.3 ± 14.3	67.4 ± 14.6	65.8 ± 14.8	64.4 ± 14.6	61.4 ± 14.0
Male, N(%)	3218 (61.2)	3236 (58.4)	3135 (57.6)	3143 (56.4)	3095 (56.2)
Caucasian, N(%)	3949 (75.1)	4211 (76.0)	4074 (74.8)	4108 (73.8)	3962 (72.0)
Body mass index, kg/m^2^	27.0 ± 9.9	27.2 ± 10.2	27.4 ± 10.0	27.6 ± 10.0	28.0 ± 9.5
Heart rate, bpm	99 ± 32	94 ± 32	93 ± 31	92 ± 31	91 ± 30
Mean blood pressure, mmHg	86 ± 42	90 ± 42	94 ± 42	96 ± 42	99 ± 40
APACHE score	50 (31–70)	45 (28–62)	42 (26–57)	40 (24–54)	36 (23–51)
Glasgow Coma Score	11 ± 5	12 ± 5	12 ± 5	12 ± 5	13 ± 5
Lab measurement					
LDL-C, mg/dL	37 ± 10	60 ± 5	79 ± 5	100 ± 8	144 ± 29
HDL-C, mg/dL	36 ± 17	41 ± 17	43 ± 16	44 ± 16	44 ± 15
Total cholesterol, mg/dL	97 ± 23	125 ± 20	146 ± 20	171 ± 21	219 ± 38
Triglyceride, mg/dL	95 (66–145)	99(71–143)	105(76–152)	113(81–160)	132(94–188)
White blood cell, *10^9^/L	10.0 (7.1–14.0)	9.4 (6.8–12.8)	9.5 (6.9–12.6)	9.4 (6.9–12.6)	9.6 (7.2–12.7)
Hemoglobin, g/dL	10.4 ± 3.1	10.9 ± 3.5	11.2 ± 3.7	11.4 ± 3.9	11.7 ± 4.2
Platelet, *10^9^/L	180 (130–240)	185 (138–237)	191 (146–242)	199 (151–247)	205 (157–252)
Albumin, g/L	27.7 ± 6.3	29.9 ± 5.9	31.1 ± 5.8	32.1 ± 5.6	33.2 ± 5.9
Primary disease of admission					
Circulatory disease, N(%)	2727 (51.9)	3489 (63.0)	3793 (69.6)	3973 (71.4)	4160 (75.6)
Respiratory disease, N(%)	573 (10.9)	475 (8.6)	401 (7.4)	347 (6.2)	233 (4.2)
Digestive disease, N(%)	414 (7.9)	239 (4.3)	155 (2.8)	111 (2.0)	94 (1.7)
Neurological disease, N(%)	211 (4.0)	230 (4.2)	206 (3.8)	255 (4.6)	231 (4.2)
Genitourinary disease, N(%)	253 (4.8)	164 (3.0)	105 (1.9)	73 (1.3)	58 (1.1)
Endocrine disease, N(%)	112 (2.1)	111 (2.0)	102 (1.9)	110 (2.0)	144 (2.6)
Pre-admission comorbidities					
ASCVD, N(%)	1960 (37.3)	1987 (35.9)	1721 (31.6)	1471 (26.4)	1422 (25.8)
Diabetes, N(%)	1655 (31.5)	1407 (25.4)	1218 (22.4)	1020 (18.3)	940 (17.1)
Hypertension, N(%)	2857 (54.3)	3096 (55.9)	3035 (55.7)	2861 (51.4)	2786 (50.6)
Heart failure, N(%)	918 (17.5)	843 (15.2)	710 (13.0)	570 (10.2)	431 (7.8)
COPD, N(%)	728 (13.8)	671 (12.1)	645 (11.8)	520 (9.3)	408 (7.4)
Renal dysfunction, N(%)	835 (15.9)	632 (11.4)	496 (9.1)	345 (6.2)	311 (5.7)
Medication					
Lipid-lowering drugs, N(%)	1869 (35.5)	1969 (35.5)	1859 (34.1)	1902 (34.2)	2116 (38.4)
Antiplatelet drugs, N(%)	647 (12.3)	633 (11.4)	527 (9.7)	504 (9.1)	423 (7.7)
Anticoagulants, N(%)	185 (3.5)	161 (2.9)	148 (2.7)	126 (2.3)	72 (1.3)
Mechanical ventilation, N(%)	1292 (24.6)	1073 (19.4)	928 (17.0)	852 (15.3)	673 (12.2)
Dialysis, N(%)	280 (5.3)	164 (3.0)	101 (1.9)	74 (1.3)	52 (0.9)
All-cause mortality, N(%)	561 (10.7)	380 (6.9)	306 (5.6)	260 (4.7)	197 (3.6)
Cardiovascular mortality, N(%)	256 (4.9)	213 (3.8)	175 (3.2)	172 (3.1)	144 (2.6)
Noncardiovascular mortality, N(%)	305 (5.8)	167 (3.0)	131 (2.4)	88 (1.6)	53 (1.0)

LDL-C, low-density lipoprotein cholesterol; HDL-C, high-density lipoprotein cholesterol; APACHE score, Acute Physiology, Age, and Chronic Health Evaluation score; ASCVD, atherosclerosis cardiovascular diseases; COPD, chronic obstructive pulmonary disease.

### Lipid levels and all-cause mortality

**Figure 1:**
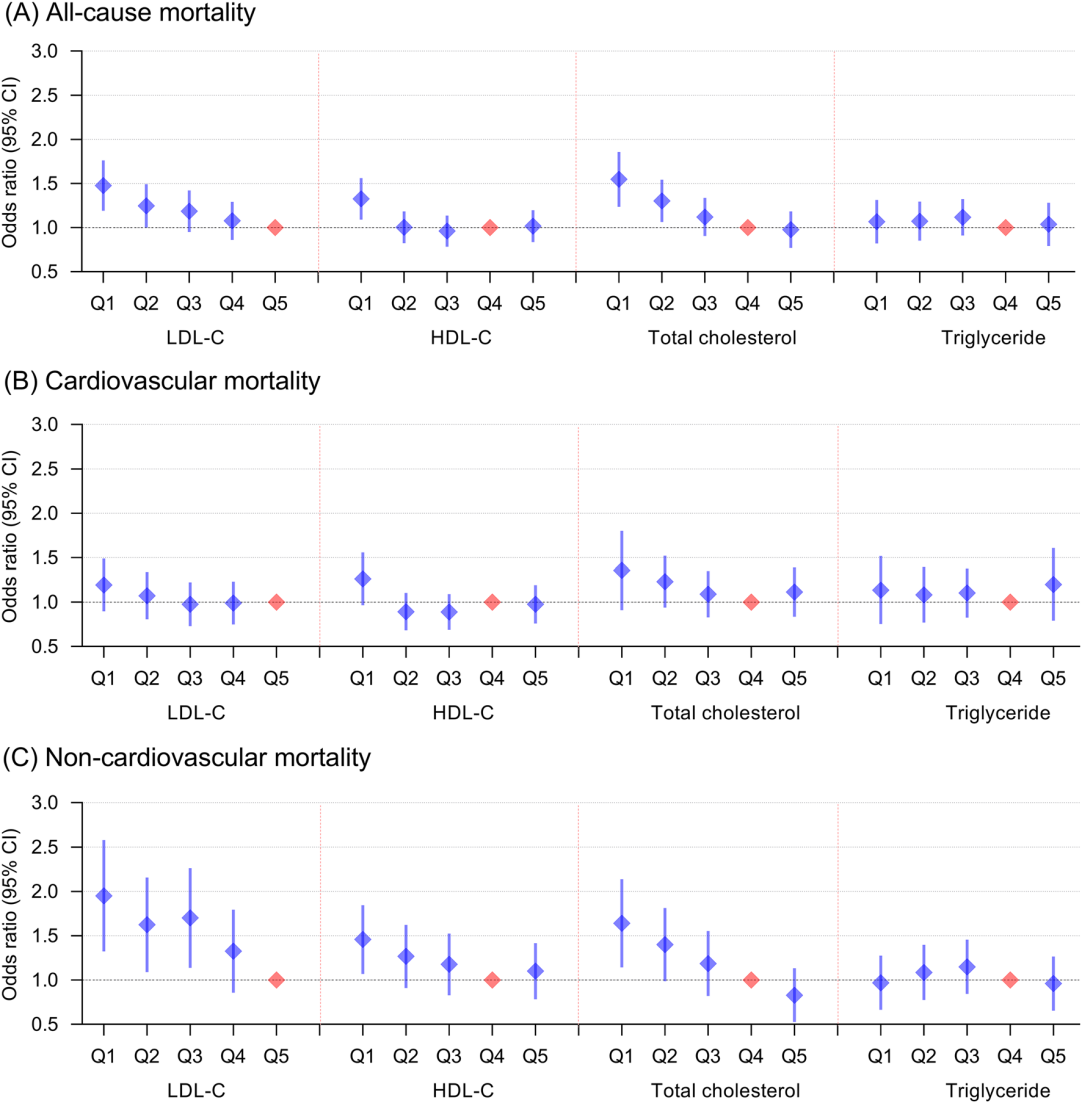
Multivariable adjusted odds ratios for all-cause and cause-specific mortality by quintiles of LDL-C, HDL-C, total cholesterol and triglyceride levels in the overall population. All-cause mortality (**A**), cardiovascular mortality (**B**) and noncardiovascular mortality (**C**) adjusted by age, sex, ethnicity, BMI, heart rate, mean blood pressure, disease severity score (APACHE score, Glasgow Coma Score), lab measurement (LDL-C, HDL-C, TC, TG, white blood cell, hemoglobin, platelet and albumin), primary disease of admission (circulatory disease, respiratory disease, digestive disease, neurological disease, genitourinary disease and endocrine disease), pre-admission comorbidities (ASCVD, diabetes, hypertension, heart failure, COPD and renal dysfunction), medication (lipid-lowering therapy, antiplatelet drug and anticoagulant), mechanical ventilation and dialysis.

**Figure 2:**
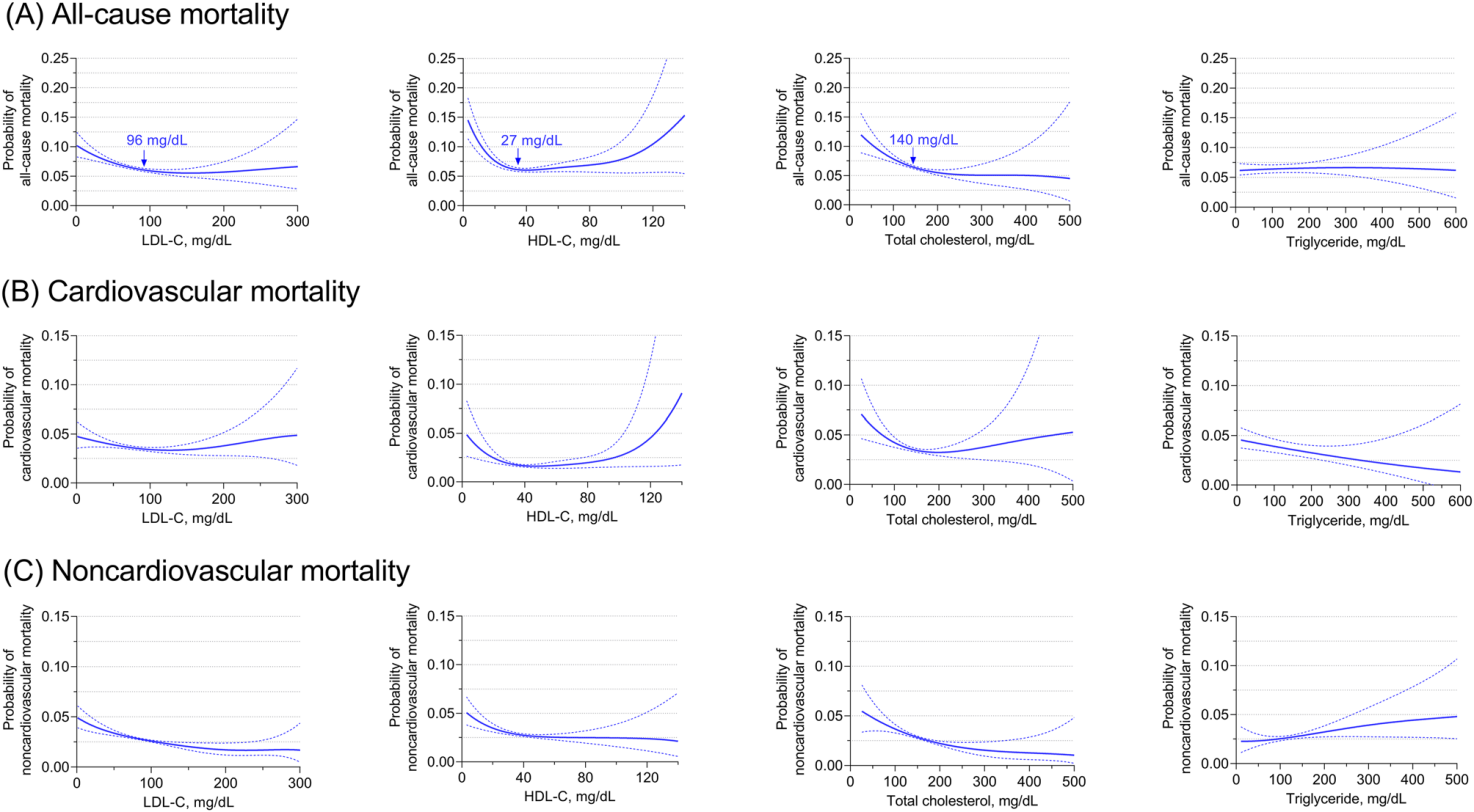
Probability of all-cause and cause-specific mortality based on LDL-C, HDL-C, total cholesterol and triglyceride levels. All-cause mortality (**A**), cardiovascular mortality (**B**) and noncardiovascular mortality (**C**) adjusted by age, sex, ethnicity, BMI, heart rate, mean blood pressure, disease severity score (APACHE score, Glasgow Coma Score), lab measurement (LDL-C, HDL-C, TC, TG, white blood cell, hemoglobin, platelet and albumin), primary disease of admission (circulatory disease, respiratory disease, digestive disease, neurological disease, genitourinary disease and endocrine disease), pre-admission comorbidities (ASCVD, diabetes, hypertension, heart failure, COPD and renal dysfunction), medication (lipid-lowering therapy, antiplatelet drug and anticoagulant), mechanical ventilation and dialysis.

**Figure 3:**
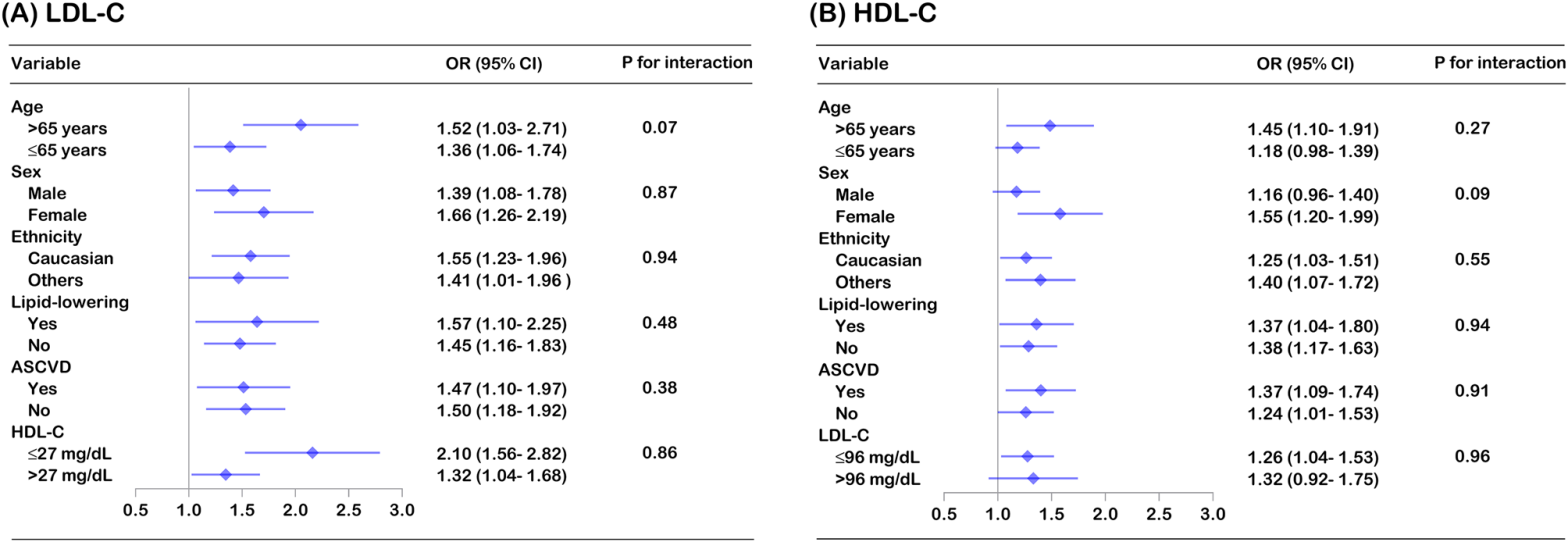
Multivariable adjusted all-cause mortality risk for LDL-C and HDL-C levels stratified by covariates. Forest plot of the odds ratio for all-cause mortality stratified by age, sex, ethnicity, lipid-lowering therapy, ASCVD, LDL-C and HDL-C levels, with the first quintile (≤ 50 mg/dL) compared to the fifth quintile of LDL-C levels (≥ 115 mg/dL) and the first quintile (≤ 28 mg/dL) compared to the fourth quintile of HDL-C levels (43–52 mg/dL). The model was adjusted by age, sex, ethnicity, BMI, heart rate, mean blood pressure, disease severity score (APACHE score, Glasgow Coma Score), lab measurement (LDL-C, HDL-C, TC, TG, white blood cell, hemoglobin, platelet and albumin), primary disease of admission (circulatory disease, respiratory disease, digestive disease, neurological disease, genitourinary disease and endocrine disease), pre-admission comorbidities (ASCVD, diabetes, hypertension, heart failure, COPD and renal dysfunction), medication (lipid-lowering therapy, antiplatelet drug and anticoagulant), mechanical ventilation and dialysis.

Compared with the fifth quintile of LDL-C (≥ 115 mg/dL), the risk of all-cause mortality in the first quintile (< 51 mg/dL; OR 1.46, 95% CI: 1.20–1.77) and the second quintile (51–69 mg/dL; OR 1.23, 95% CI: 1.01–1.50) increased significantly. Compared with the fourth quintile of TC levels (159–188 mg/dL), the first quintile (< 110 mg/dL; OR 1.52, 95% CI: 1.25–1.87) and the second quintile (111–135 mg/dL; OR 1.29, 95% CI: 1.07–1.55) also had a higher risk of all-cause mortality. For HDL-C, only those in the first quintile (< 29 mg/dl; OR 1.31, 95% CI: 1.10–1.57) had a greater risk of all-cause mortality than those in the fourth quintile of HDL-C levels (43–52 mg/dL). Additionally, we did not observe any association between TG quintile and all-cause mortality (Fig. 1, eTable 4). Significantly, the findings were consistent in individuals with and without lipid-lowering therapy, although the confidence interval was wider in individuals using lipid-lowering drugs (eFigure 1, eFigure 2). Using an adjusted nonlinear curve, a J-shaped association between LDL-C, HDL-C, TC levels and outcomes was evident (Fig. 2). No significant interaction was found for LDL-C, HDL-C, or TC on all-cause mortality with predefined strata of age, sex, ethnicity, lipid-lowering therapy, ASCVD, LDL-C and HDL-C (Fig. 3, eFigure 3).

### Lipid levels and cause-specific mortality

According to the quintiles of LDL-C, HDL-C, TC and TG, there was no significant difference in cardiovascular mortality. Compared to the fifth quintile of LDL-C, the first to third quintiles were associated with an increased risk of noncardiovascular mortality. Compared to the fourth quintile of TC, the first and second quintiles were also related to a higher risk of noncardiovascular mortality. For HDL-C, only the first quintile had an increased risk of noncardiovascular mortality compared to the reference quintile (Fig. 1, eTable 5, eTable 6). The findings for cause-specific mortality were similar in those with and without lipid-lowering treatment (eFigure 1, eFigure 2). Using an adjusted nonlinear curve, there was a significant J-shaped association between LDL-C, HDL-C, and TC levels and noncardiovascular mortality but no significant association with cardiovascular mortality (Fig. 2).

### Risk inflection point of lipid concentration for all-cause mortality

In multivariable adjusted analysis, the risk inflection point of lipid concentration for all-cause mortality was 96 mg/dL for LDL-C, 27 mg/dL for HDL-C and 140 mg/dL for TC. Individuals with LDL-C, HDL-C and TC levels below the risk inflection point accounted for 66.9%, 16.6% and 44.4%, respectively. Before the corresponding risk inflection points, the risk of all-cause death decreased by 8% for every 10 mg/dL increase in LDL-C, by 36% for every 10 mg/dL increase in HDL-C and by 9% for every 10 mg/dL increase in TC (eTable 7), and the risk of all-cause mortality plateaued after the inflection points.

### Synergistic effects of low lipid levels on mortality

**Figure 4:**
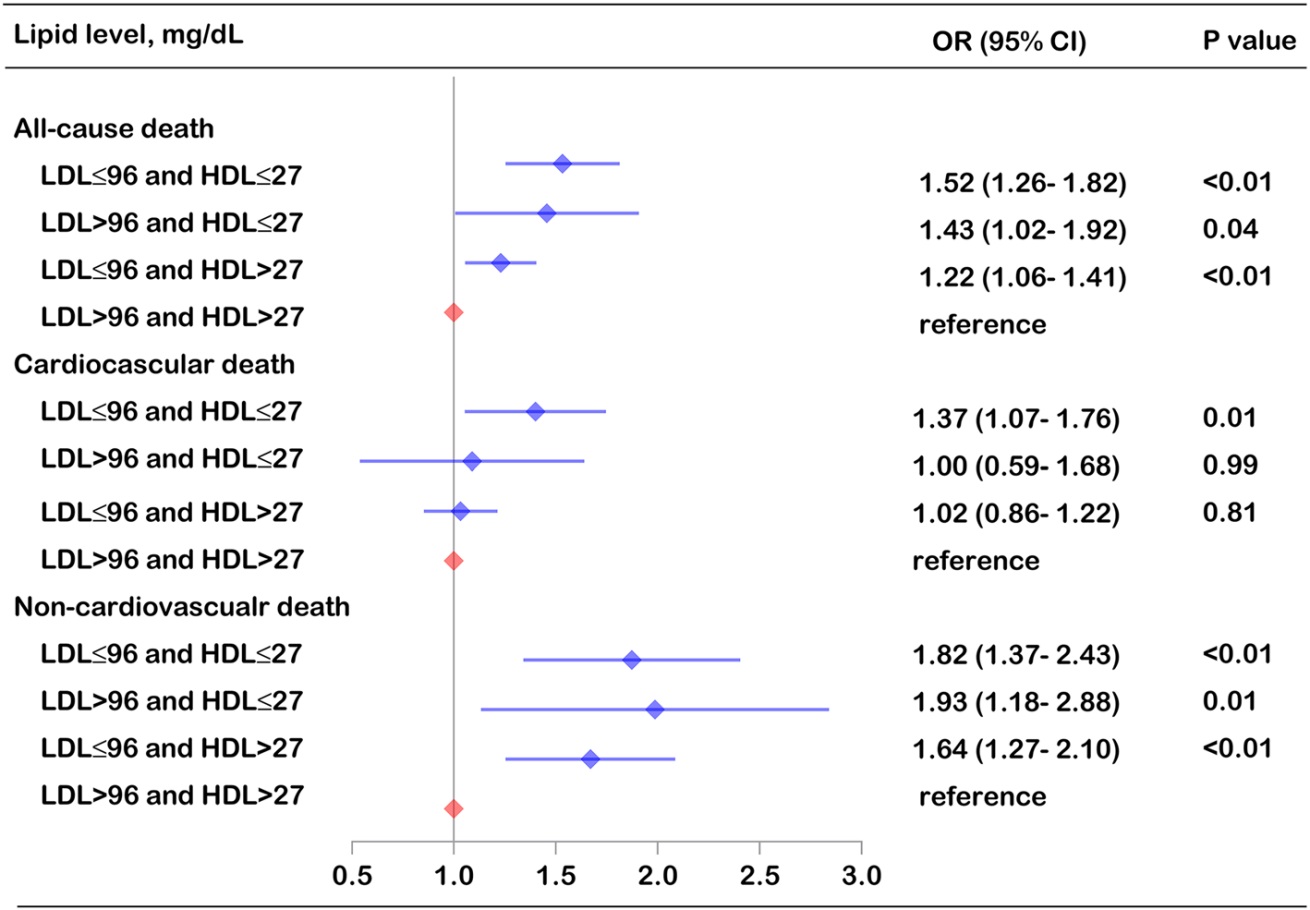
Multivariable adjusted all-cause and cause-specific mortality risk according to the risk inflection point of LDL-C and HDL-C. The model was adjusted by age, sex, ethnicity, BMI, heart rate, mean blood pressure, disease severity score (APACHE score, Glasgow Coma Score), lab measurement (LDL-C, HDL-C, TC, TG, white blood cell, hemoglobin, platelet and albumin), primary disease of admission (circulatory disease, respiratory disease, digestive disease, neurological disease, genitourinary disease and endocrine disease), pre-admission comorbidities (ASCVD, diabetes, hypertension, heart failure, COPD and renal dysfunction), medication (lipid-lowering therapy, antiplatelet drug and anticoagulant), mechanical ventilation and dialysis.

The percentage of individuals according to the joint categories of LDL-C and HDL-C ranged from 2.9 to 53.3%. Those with LDL-C ≤ 96 and HDL-C > 27 mg/dL accounted for the largest proportion, and those with LDL-C > 96 and HDL-C ≤ 27 mg/dL accounted for the smallest proportion. Compared with individuals with LDL-C > 96 and HDL-C > 27 mg/dL, those with LDL-C ≤ 96 and HDL-C ≤ 27 mg/dL had the highest all-cause mortality (OR 1.52, 95% CI: 1.26–1.82) and noncardiovascular mortality (OR 1.82, 95% CI: 1.37–2.43), followed by those with LDL-C > 96 and HDL-C ≤ 27 mg/dL and those with LDL-C ≤ 96 and HDL-C > 27 mg/dL. Meanwhile, individuals with LDL-C ≤ 96 and HDL-C ≤ 27 mg/dL also had a higher cardiovascular mortality (OR 1.37, 95% CI: 1.07–1.76) than those with LDL-C > 95 and HDL-C > 27 mg/dL (Fig. 4, eTable 8).

### Sensitivity analysis

The J-shaped association between lipid levels and all-cause death was consistent when we excluded individuals dying within 24 h of ICU entry (eFigure 4, eTable 9). The main results were not altered by missing data with covariates when a complete-case analysis was performed (eFigure 5, eTable 10). Similar results were also obtained using the first measurement of lipid levels after ICU admission instead of the baseline measurement at hospital admission (eFigure 6, eTable 11). Last, estimating cumulative incidence using the Kaplan‒Meier estimator, individuals with the lowest LDL-C, HDL-C or TC levels (first quintile) had the worst cumulative survival (eFigure 7). Compared with individuals with LDL-C > 96 and HDL-C > 27 mg/dL, those with LDL-C ≤ 96 and HDL-C ≤ 27 mg/dL had worse survival (eFigure 8).

## Discussion

In this large cohort study of ICU patients, we found a J-shaped association between LDL-C, HDL-C and TC levels and all-cause and noncardiovascular mortality. Lower concentrations were associated with a higher risk of all-cause mortality, with a significant inflection point in risk at 96 mg/dL for LDL-C, 27 mg/dL for HDL-C and 140 mg/dL for TC. Thus, low LDL-C combined with low HDL-C has a magnifying effect on the risk of mortality. These findings advance our understanding of the complex association between lipid levels and clinical outcomes, and they highlight the potentially harmful effects of low LDL-C, HDL-C and TC levels in critically ill patients, particularly low LDL-C incorporated with low HDL-C.

Epidemiological studies have shown that there is a lipid paradox in different populations. For example, an observational study revealed that a low TC concentration was correlated with a greater risk of death caused by cancer, trauma and respiratory disease^16,17^. This paradoxical correlation of TC with mortality was also verified in elderly people, with high TC levels contributing to long-term survival^18,19^. Meanwhile, some evidence has linked low LDL-C to an increased risk of all-cause mortality and cardiovascular and cancer mortality and reported that the benefits of lipid-lowering therapy diminished with advancing age^10,20,21^. Similar findings were demonstrated for HDL-C, with a significant U-shape association between HDL-C and all-cause mortality^8,9^. In this study, we found a J-shape correlation between LDL-C, HDL-C, TC and mortality risk in critically ill patients, which is in agreement with previous studies.

Although numerous studies have confirmed that LDL-C is the leading cause of developing ASCVD and that lipid-lowering therapy is the cornerstone for preventing ASCVD progression and improving long-term survival, the relationship between LDL-C level and mortality risk in different populations remains controversial. In this study, the association between LDL-C level and all-cause mortality was nonlinear, with a risk inflection point of approximately 96 mg/dL. The risk of death decreased by 8% for every 10 mg/dL increase in LDL-C before the risk inflection point and then leveled off. A similar association was found with noncardiovascular mortality but not with cardiovascular mortality. Similar to the current findings, the MONDO study found that LDL-C was inversely associated with all-cause and infectious mortality in patients with end-stage renal disease but not with cardiovascular mortality^22^. A large prospective cohort study of the general population in Denmark also found that low LDL-C was closely related to increased all-cause, cancer and other-cause mortality but not to cardiovascular mortality^7^. Consistent results were found with TC, and individuals in the lowest quintile of TC had the highest risk of all-cause and noncardiovascular mortality after adjusting for a series of potential confounding factors. The results of HDL-C are in line with the published data of cardiovascular patients, and low HDL-C levels are correlated with a higher risk of all-cause mortality. Although the TG paradox has been reported^12,23^, we did not find a significant inverse association between TGs and mortality risk. Additionally, we did not find any difference in the association between lipid levels and mortality among individuals with or without lipid-lowering treatment.

Although we did not find a correlation between LDL-C and HDL-C alone with cardiovascular death, we found a significant synergistic effect of low LDL-C and HDL-C on cardiovascular mortality. Therefore, the disadvantage of low HDL-C may not be offset by the advantage of low LDL, although low LDL is a theoretically protective factor for cardiovascular death. These findings suggest that clinicians should pay close attention to low LDL-C, HDL-C and TC levels, which are important predictors of high mortality risk in critically ill patients. More importantly, since the potential risk of a combination of low LDL-C and low HDL-C has not been well studied and appreciated, special attention should be given to this high-risk group. Careful monitoring of signs of health deterioration and more timely treatment adjustments are necessary. On the other hand, 31.3% of individuals had a history of ASCVD, and 35.5% of individuals were taking lipid-lowering drugs (statins accounted for 99%) in this ICU cohort. Some of them were not admitted due to cardiovascular emergencies (such as acute myocardial infarction), and the effect of intensive lipid-lowering treatment on the prognosis of these patients needs further study.Several possible reasons could explain the adverse effects of low lipid levels. Cholesterol is an indispensable component of cell membranes and plays a crucial role in intracellular signal pathway transduction. In addition, it is a substrate for steroid hormone synthesis, which is beneficial for resisting fatal stress. LDL-C also has a protective effect on host defense against multiple pathogens, and extremely low LDL-C concentrations may increase susceptibility to life-threatening diseases^24^. HDL-C has more complex proteomic characteristics and therefore has pleiotropic biological effects, including cholesterol reverse transport, anti-inflammatory, anti-oxidative and anti-apoptosis roles^25^. Due to the positive protective role of LDL-C, HDL-C and TC, low circulating concentrations may have adverse effects on survival.

Several limitations need to be considered. First, due to the observational design of this study, we could not address causality, and this problem can be clarified in the future by Mendelian random analysis. Second, although we extensively adjusted for potential confounders, including demographic characteristics, admission diagnosis, major comorbidities, critical illness score and treatments, the impact of residual and unmeasured confounding could not be completely excluded. Third, this study only included critically ill patients in the ICU, and the findings may not be extended to non-ICU patients. Fourth, the patient selection for inclusion in the database was based on a method of stratified random sample extraction; thus, these results may not be extrapolated to ICUs elsewhere with different resources or practices. Further studies are warranted to confirm these findings.

## Conclusion

This study found a J-shaped association between lipid levels and mortality risk, with low LDL-C, TC and HDL-C levels related to increased all-cause and noncardiovascular death in critically ill patients. More notably, low LDL-C combined with low HDL-C has a significant synergistic effect on greater all-cause and cause-specific mortality.

## Supplementary Information

Supplementary Information.

## Data Availability

The database used in the current study is available from https://www.physionet.org/content/eicu-crd-demo/2.0.1/.
