# Supplementary material for: Association between lipid levels and all-cause and cause-specific mortality in critically ill patients
Source: Sci Rep. 2023 Mar 29;13:5109. doi: 10.1038/s41598-023-32209-z (PMC10060260; doi:10.1038/s41598-023-32209-z)

Supplementary material

Association between lipid levels and all-cause and cause-specific mortality in critically ill patients

Shan Li, MD; Wei Zhang; MD, Hongbin Liu*, MD
[1]Department of Cardiology, The Second Medical Center, Chinese People Liberation Army General Hospital, Beijing, China, [2]National Clinical Research Center for Geriatric Disease, Beijing, China.
*Corresponding author. Email: liuhbinpla@163.com

Legends

eTable 1. Baseline Characteristics of individuals according to quintiles of HDL-C levels

eTable 2. Baseline Characteristics of individuals according to quintiles of total cholesterol levels

eTable 3. Baseline Characteristics of individuals according to quintiles of triglyceride levels

eTable 4. Unadjusted and adjusted odds ratios for all-cause mortality by quintiles of LDL-C, HDL-C, total cholesterol and triglyceride levels

eTable 5. Unadjusted and adjusted odds ratios for cardiovascular mortality by quintiles of LDL-C, HDL-C, total cholesterol and triglyceride levels

eTable 6. Unadjusted and adjusted odds ratios for non-cardiovascular mortality by the quintiles of LDL-C, HDL-C, total cholesterol and triglyceride levels

eTable 7. Univariable and multivariable analysis for all-cause mortality with lipid levels below or above the risk inflection point

eTable 8. Unadjusted and adjusted odds ratios for all-cause mortality by LDL-C level combined with HDL-C level

eTable 9. Unadjusted and adjusted odds ratios for all-cause mortality according to quintiles of LDL-C, HDL-C, total cholesterol and triglyceride levels by excluding death within the first 24 hours of ICU entry

eTable 10. Unadjusted and adjusted odds ratios for all-cause mortality according to quintiles of LDL-C, HDL-C, total cholesterol and triglyceride levels with complete-case analyse

eTable 11. Unadjusted and adjusted odds ratios for all-cause mortality according to quintiles of LDL-C, HDL-C, total cholesterol and triglyceride levels by using the first measurement of ICU admission

eFigure 1. Multivariable adjusted odds ratios for all-cause and cause-specific mortality by quintiles of LDL-C, HDL-C, total cholesterol and triglyceride levels in individuals with lipid-lowering therapy

eFigure 2. Probability of all cause and cause-specific mortality based on LDL-C, HDL-C, total cholesterol and triglyceride levels in individuals without lipid-lowering therapy

eFigure 3. Multivariable adjusted all-cause mortality risk for total cholesterol levels stratified by covariates

eFigure 4. Probability of all cause mortality based on LDL-C, HDL-C, total cholesterol and triglyceride levels by excluding death within the first 24 hours of ICU entry

eFigure 5. Probability of all cause mortality based on LDL-C, HDL-C, total cholesterol and triglyceride levels with complete-case analyse

eFigure 6. Probability of all cause mortality based on LDL-C, HDL-C, total cholesterol and triglyceride levels by using the first measurement of ICU admission

eFigure 7. Kaplan–Meier curve for cumulative incidence with all cause mortality by quintiles of lipid levels

eFigure 8. Kaplan–Meier curve for cumulative incidence with all cause mortality by LDL-C level combined with HDL-C level

eTable 1. Baseline Characteristics of individuals according to quintiles of HDL-C levels

Baseline HDL-C	1st Quintile ≤28mg/dL	2nd Quintile 29-36mg/dL	3rd Quintile 36-42mg/dL	4th Quintile 43-52mg/dL	5th Quintile ≥53mg/dL
N(%)	5136 (18.8)	5410 (19.8)	5517 (20.2)	5590 (20.5)	5663 (20.7)
Age, years	62.7 ± 14.7	64.1 ± 14.2	65.4 ± 14.3	66.7 ± 14.6	68.0 ± 14.9
Male, N(%)	3526 (68.7)	3719 (68.7)	3447 (62.5)	2965 (53.0)	2170 (38.3)
Caucasian, N%()	3896 (75.9)	4091 (75.6)	4127 (74.8)	4066 (72.7)	4124 (72.8)
Body mass index, kg/m^2	28.3 ± 10.3	28.5 ± 10.2	27.8 ± 9.9	27.0 ± 9.8	25.7 ± 9.1
Heart rate, bpm	101 ± 31	92 ± 31	91 ± 31	92 ± 31	94 ± 31
Mean blood pressure, mmHg	86 ± 42	92 ± 41	94 ± 41	96 ± 42	98 ± 43
APACHE score	47(27-67)	41 (24-57)	41 (25-56)	41 (25-56)	42 (26-57)
Glasgow Coma Score	12 ± 5	12 ± 5	12 ± 5	12 ± 5	12 ± 5
Lab measurement					
LDL-C, mg/dL	69 ± 37	84 ± 38	89 ± 39	91 ± 40	89 ± 39
HDL-C, mg/dL	21 ± 6	32 ± 2	39 ± 2	47 ± 3	66 ± 13
Total cholesterol, mg/dL	123 ± 48	146 ± 45	155 ± 46	160 ± 45	173 ± 44
Triglyceride, mg/dL	137(95-198)	127(91-185)	115(83-163)	99 (73-138)	81(60-112)
White blood cell, *10^9/L	10.5 (7.5-14.8)	9.7 (7.1-12.9)	9.5 (7.2-12.6)	9.3 (6.9-12.4)	9.0 (6.4-12.2)
Hemoglobin, g/dL	10.8 ± 3.40	11.2 ± 3.8	11.4 ± 3.7	11.2 ± 3.8	11.0 ± 3.8
Platelet, *10^9/L	185 (133-249)	192 (144-242)	194 (148-242)	194 (146-243)	196 (146-245)
Albumin, g/L	27.5 ± 6.7	30.8 ± 6.0	31.5 ± 5.7	31.8 ± 5.8	32.1 ± 5.7
Primary disease of admission					
Circulatory disease, N(%)	2673 (52.0)	3688 (68.2)	4012 (72.7)	3927 (70.3)	3842 (67.8)
Respiratory disease, N(%)	586 (11.4)	349 (6.5)	307 (5.6)	355 (6.4)	432 (7.6)
Digestive disease, N(%)	410 (8.0)	230 (4.3)	134 (2.4%)	120 (2.1)	119 (2.1)
Neurological disease, N(%)	177 (3.4)	193 (3.6)	193 (3.5)	266 (4.8)	304 (5.4)
Genitourinary disease, N(%)	281 (5.5)	109 (2.0)	96 (1.7)	98 (1.8)	69 (1.2)
Endocrine disease, N(%)	129 (2.5)	105 (1.9)	107 (1.9)	113 (2.0)	125 (2.2)
Pre-admission comorbidities					
ASCVD, N(%)	1621 (31.6)	1859 (34.4)	1821 (33.0)	1711 (30.6)	1549 (27.4)
Diabetes, N(%)	1401 (27.3)	1446 (26.7)	1302 (23.6)	1121 (20.1)	970 (17.1)
Hypertension, N(%)	2685 (52.3)	2965 (54.8)	2988 (54.2)	3003 (53.7)	2994 (52.9)
Heart failure, N(%)	776 (15.1)	732 (13.5)	650 (11.8)	640 (11.4)	674 (11.9)
COPD, N(%)	566 (11.0)	549 (10.1)	542 (9.8)	553 (9.9)	762 (13.5)
Renal failure, N(%)	608 (11.8)	557 (10.3)	513 (9.3)	481 (8.6)	460 (8.1)
Medication					
Lipid-lowering drugs, N(%)	1618 (31.5)	2115 (39.1)	2147 (38.9)	2028 (36.3)	1807 (31.9)
Antiplatelet drugs, N(%)	542 (10.6)	578 (10.7)	605 (11.0)	497 (8.9)	512 (9.0)
Anticoagulants, N(%)	161 (3.1)	126 (2.3)	148 (2.7)	135 (2.4)	122 (2.2)
Mechanical ventilation, N(%)	1148 (22.4)	945 (17.5)	902 (16.3)	895 (16.0)	928 (16.4)
Dialysis, N(%)	162 (3.2)	139 (2.6)	118 (2.1)	121 (2.2)	131 (2.3)
All-cause mortality, N(%)	479 (9.3)	302 (5.6)	283 (5.1)	306 (5.5)	334 (5.9)

Cardiovascular mortality, N(%)	204 (4.0)	164 (3.0)	177 (3.2)	200 (3.6)	215 (3.8)
Noncardiovascular mortality, N(%)	275 (5.4)	138 (2.6)	106 (1.9)	106 (1.9)	119 (2.1)

LDL-C, low-density lipoprotein cholesterol; HDL-C, high-density lipoprotein cholesterol; APACHE score, Acute Physiology, Age, and Chronic Health Evaluation score; ASCVD, atherosclerosis cardiovascular disease; COPD, chronic obstructive pulmonary disease.

eTable 2. Baseline Characteristics of individuals according to quintiles of total cholesterol levels

Baseline TC	1st Quintile ≤110mg/dL	2nd Quintile 111-135 mg/dL	3rd Quintile 136-158 mg/dL	4th Quintile 159-188 mg/dL	5th Quintile ≥189mg/dL
N(%)	5270 (19.3)	5626 (20.6)	5328 (19.5)	5609 (20.5)	5483 (20.1)
Age, years	68.1 ± 14.5	67.6 ± 14.5	66.1 ± 14.7	64.3 ± 14.5	61.3 ± 14.2
Male, N(%)	3421 (64.9)	3489 (62.0)	3000 (56.3)	3089 (55.1)	2828 (51.6)
Caucasian, N%()	3922 (74.4)	4264 (75.8)	4028 (75.6)	4124 (73.5)	3966 (72.3)
Body mass index, kg/m2	27.0 ± 10.1	27.3 ± 10.1	27.2 ± 10.1	27.9 ± 9.7	27.8 ± 9.6
Heart rate, bpm	100 ± 32	94± 32	92 ± 31	91 ± 31	92 ± 30
Mean blood pressure, mmHg	84 ± 42	91 ± 42	93 ± 41	97 ± 42	100 ± 40
APACHE score	51(31-69)	45(28-62)	42(25-57)	39(24-54)	36(23-50)
Glasgow Coma Score	11 ± 5	12 ± 5	12 ± 5	12 ± 5	12 ± 5
Lab measurement					
LDL-C, mg/dL	41 ± 14	62 ± 14	79 ± 15	99 ± 17	140 ± 34
HDL-C, mg/dL	30 ± 13	39 ± 13	43 ± 15	46 ± 16	48 ± 19
Total cholesterol, mg/dL	91 ± 15	124 ± 7	147 ± 7	173 ± 9	223 ± 36
Triglyceride, mg/dL	88(64-122)	98(72-138)	106(76-151)	119(84-170)	146(100-215)
White blood cell, *10^9/L	10.1 (7.1-14.4)	9.6 (7.0-12.9)	9.4 (6.8-12.5)	9.3 (6.9-12.5)	9.6 (7.1-12.6)
Hemoglobin, g/dL	10.4 ± 3.0	11.0 ± 3.3	11.2 ± 3.8	11.4 ± 4.0	11.6 ± 4.2
Platelet, *10^9/L	180 (128-242)	186 (140-238)	193 (146-242)	199 (151-244)	204 (155-252)
Albumin, g/L	27.2 ± 6.3	30.1 ± 5.8	31.4 ± 5.6	32.4 ± 5.6	33.3 ± 6.0
Primary disease of admission					
Circulatory disease, N(%)	2648 (50.2)	3652 (64.9)	3752 (70.4)	4025 (71.8)	4065 (74.1)
Respiratory disease, N(%)	625 (11.9)	462 (8.2)	369 (6.9)	330 (5.9)	243 (4.4)
Digestive disease, N(%)	437 (8.3)	243 (4.3)	141 (2.6)	91 (1.6)	101 (1.8)
Neurological disease, N(%)	199 (3.8)	224 (4.0)	210 (3.9)	251 (4.5)	249 (4.5)
Genitourinary disease, N(%)	284 (5.4)	143 (2.5)	100 (1.9)	73 (1.3)	53 (1.0)
Endocrine disease, N(%)	98 (1.9)	104 (1.8)	89 (1.7)	123 (2.2)	165 (3.0)
Pre-admission comorbidities					
ASCVD, N(%)	1970 (37.4)	2001 (35.6)	1693 (31.8)	1494 (26.6)	1403 (25.6)
Diabetes, N(%)	1539 (29.2)	1457 (25.9)	1183 (22.2)	1062 (18.9)	999 (18.2)
Hypertension, N(%)	2826 (53.6)	3185 (56.6)	2892 (54.3)	2948 (52.6)	2784 (50.8)
Heart failure, N(%)	1010 (19.2)	856 (15.2)	616 (11.6)	562 (10.0)	428 (7.8)
COPD, N(%)	696 (13.2)	683 (12.1)	609 (11.4)	527 (9.4)	457 (8.3)
Renal failure, N(%)	841 (16.0)	625 (11.1)	458 (8.6)	379 (6.8)	316 (5.8)

Medication					
Lipid-lowering drugs, N(%)	1797 (34.1)	1998 (35.5)	1882 (35.3)	1954 (34.8)	2084 (38.0)
Antiplatelet drugs, N(%)	669 (12.7)	635 (11.3)	506 (9.5)	502 (8.9)	422 (7.7)
Anticoagulants, N(%)	202 (3.8)	178 (3.2)	138 (2.6)	104 (1.9)	70 (1.3)
Mechanical ventilation, N(%)	1268 (24.1)	1098 (19.5)	908 (17.0)	833 (14.9)	711 (13.0)
Dialysis, N(%)	261 (5.0)	165 (2.9)	102 (1.9)	82 (1.5)	61 (1.1)
All-cause mortality, N(%)	564 (10.7)	392 (7.0)	292 (5.5)	250 (4.5)	206 (3.8)
Cardiovascular mortality, N(%)	244 (4.6)	223 (4.0)	183 (3.4)	161 (2.9)	149 (2.7)
Noncardiovascular mortality, N(%)	320 (6.1)	169 (3.0)	109 (2.0)	89 (1.6)	57 (1.0)

LDL-C, low density-lipoprotein cholesterol; HDL-C, high-density lipoprotein cholesterol; APACHE score, Acute Physiology, Age, and Chronic Health Evaluation score; ASCVD, atherosclerosis cardiovascular disease; COPD, chronic obstructive pulmonary disease.

eTable 3. Baseline Characteristics of individuals according to quintiles of triglyceride levels

Baseline TG	1st Quintile ≤70mg/dL	2nd Quintile 71-94mg/dL	3rd Quintile 95-124mg/dL	4th Quintile 125-174mg/dL	5th Quintile ≥175mg/dL
N(%)	5444 (19.9)	5332 (19.5)	5578 (20.4)	5470 (20.0)	5492 (20.1)
Age, years	68.6 ± 15.3	68.0 ± 14.6	66.4 ± 14.0	63.9 ± 14.1	60.4 ± 13.7
Male, N(%)	3155 (58.0)	3038 (57.0)	3222 (57.8)	3149 (57.6)	3263 (59.4)
Caucasian, N%()	3933 (72.2)	3957 (74.2)	4168 (74.7)	4070 (74.4)	4176 (76.0)
Body mass index, kg/m2	25.4 ± 9.3	26.4 ± 9.8	27.4 ± 9.8	28.5 ± 10.0	29.4 ± 10.1
Heart rate, bpm	94 ± 32	94 ± 32	94 ± 31	94 ± 31	93 ± 30
Mean blood pressure, mmHg	91 ± 42	92 ± 42	93 ± 42	95 ± 42	95 ± 42
APACHE score	44 (28-60)	44 (27-60)	42 (26-59)	41 (24-57)	39 (23-56)
Glasgow Coma Score	12 ± 5	12 ± 5	12 ± 5	12 ± 5	12 ± 5
Lab measurement					
LDL-C, mg/dL	72 ± 33	80 ± 35	85 ± 37	91 ± 41	94 ± 46
HDL-C, mg/dL	50 ± 19	45± 17	41 ± 15	38 ± 14	34 ± 12
Total cholesterol, mg/dL	134 ± 42	141 ±42	148 ± 43	158 ± 47	178 ± 54
Triglyceride, mg/dL	55 ± 11	82 ± 7	109 ± 9	147 ± 14	251 ± 81
White blood cell, $*10^9$/L	9.0 (6.5-12.3)	9.5 (7.0-12.8)	9.6 (7.1-12.9)	9.8 (7.2-13.1)	9.9 (7.2-13.3)
Hemoglobin, g/dL	11.0 ± 3.5	11.0 ± 3.6	11.2 ± 3.7	11.2 ± 4.0	11.3 ± 4.0
Platelet, $*10^9$/L	185 (137-236)	193 (142-245)	195 (144-248)	197 (148-246)	194 (146-245)
Albumin, g/L	30.4 ± 6.0	30.5 ± 6.2	30.5 ± 6.1	30.6 ± 6.4	31.1 ± 6.6
Primary disease of admission					
Circulatory disease, N(%)	3535 (64.9)	3570 (67.0)	3766 (67.5)	3621 (66.2)	3650 (66.5)
Respiratory disease, N(%)	529 (9.7)	425 (8.0)	370 (6.6)	381 (7.0)	324 (5.9)
Digestive disease, N(%)	185 (3.4)	190 (3.6)	220 (3.9)	214 (3.9)	204 (3.7)
Neurological disease, N(%)	239 (4.4)	238 (4.5)	239 (4.3)	219 (4.0)	198 (3.6)
Genitourinary disease, N(%)	120 (2.2)	100 (1.9)	123 (2.2)	150 (2.7)	160 (2.9)
Endocrine disease, N(%)	81 (1.5)	68 (1.3)	88 (1.6)	132 (2.4)	210 (3.8)
Pre-admission comorbidities					

ASCVD, N(%)	1674 (30.7)	1679 (31.5)	1850 (33.2)	1639 (30.0)	1719 (31.3)
Diabetes, N(%)	946 (17.4)	1011 (19.0)	1245 (22.3)	1356 (24.8)	1682 (30.6)
Hypertension, N(%)	2821 (51.8)	2874 (53.9)	2991 (53.6)	2997 (54.8)	2952 (53.8)
Heart failure, N(%)	843 (15.5)	794 (14.9)	724 (13.0)	614 (11.2)	497 (9.0)
COPD, N(%)	693 (12.7)	616 (11.6)	598 (10.7)	570 (10.4)	495 (9.0)
Renal failure, N(%)	546 (10.0)	534 (10.0)	520 (9.3)	502 (9.2)	517 (9.4)
Medication					
Lipid-lowering drugs, N(%)	1768 (32.5)	1840 (34.5)	1935 (34.7)	2031 (37.1)	2141 (39.0)
Antiplatelet drugs, N(%)	570 (10.5)	544 (10.2)	570 (10.2)	511 (9.3)	539 (9.8)
Anticoagulants, N(%)	178 (3.3)	167 (3.1)	147 (2.6)	123 (2.2)	77 (1.4)
Mechanical ventilation, N(%)	945 (17.4)	925 (17.3)	980 (17.6)	987 (18.0)	981 (17.9)
Dialysis, N(%)	144 (2.6)	123 (2.3)	139 (2.5)	125 (2.3)	140 (2.5)
All-cause mortality, N(%)	371 (6.8)	362 (6.8)	360 (6.5)	313 (5.7)	298 (5.4)
Cardiovascular mortality, N(%)	219 (4.0)	205 (3.8)	203 (3.6)	168 (3.1)	165 (3.0)
Non-cardiovascular mortality, N(%)	152 (2.8)	157 (2.9)	157 (2.8)	145 (2.7)	133 (2.4)

LDL-C, low-density lipoprotein cholesterol; HDL-C, high-density lipoprotein cholesterol; APACHE score, Acute Physiology, Age, and Chronic Health Evaluation score; ASCVD, atherosclerosis cardiovascular disease; COPD, chronic obstructive pulmonary disease.

eTable 4. Unadjusted and adjusted odds ratios for all-cause mortality by quintiles of LDL-C, HDL-C, total cholesterol and triglyceride levels

All-cause mortality	Quintile 1	Quintile 2	Quintile 3	Quintile 4	Quintile 5
	OR (95% CI)	OR (95% CI)	OR (95% CI)	OR (95% CI)	OR (95% CI)
**LDL-C**					
Model I	3.22 (2.72, 3.80)	1.98 (1.66, 2.37)	1.60 (1.34, 1.93)	1.32 (1.09, 1.59)	reference
Model II	2.75 (2.32, 3.26)	1.72 (1.44, 2.05)	1.44 (1.20, 1.73)	1.22 (1.01, 1.48)	reference
Model III	1.46 (1.20, 1.77)	1.23 (1.01, 1.50)	1.17 (0.96, 1.43)	1.06 (0.87, 1.30)	reference
**HDL-C**					
Model I	1.78 (1.53, 2.06)	1.02 (0.87, 1.20)	0.93 (0.79, 1.10)	reference	1.08 (0.92, 1.27)
Model II	2.06 (1.77, 2.39)	1.13 (0.96, 1.33)	0.99 (0.83, 1.17)	reference	1.02 (0.87, 1.20)
Model III	1.31 (1.10, 1.57)	0.99 (0.83, 1.19)	0.95 (0.79, 1.14)	reference	1.01 (0.84, 1.20)
**Total cholesterol**					
Model I	2.57 (2.20, 3.00)	1.61 (1.36, 1.89)	1.24 (1.05, 1.48)	reference	0.84 (0.69, 1.01)
Model II	2.40 (2.06, 2.81)	1.50 (1.27, 1.77)	1.19 (1.00, 1.42)	reference	0.90 (0.74, 1.08)
Model III	1.52 (1.25, 1.87)	1.29 (1.07, 1.55)	1.11 (0.91, 1.34)	reference	0.96 (0.78, 1.19)
**Triglyceride**					
Model I	1.20 (1.03, 1.41)	1.20 (1.03, 1.40)	1.14 (0.97, 1.33)	reference	0.95 (0.80, 1.11)
Model II	1.05 (0.89, 1.22)	1.06 (0.91, 1.25)	1.06 (0.91, 1.24)	reference	1.05 (0.89, 1.23)
Model III	1.05 (0.83, 1.32)	1.06 (0.86, 1.30)	1.10 (0.92, 1.33)	reference	1.02 (0.80, 1.29)

Model I, unadjusted model; Model II, all cause mortality adjusted by age, sex and ethnicity; Model III, all cause mortality adjusted by age, sex, ethnicity, body mass index, heart rate, mean blood pressure, disease severity score (APACHE score and Glasgow coma score), lab measurements (LDL-C, HDL-C, total cholesterol, triglyceride, white blood cell, hemoglobin, platelet and albumin), primary disease of admission (circulatory disease, respiratory

disease, digestive disease, neurological disease, genitourinary disease and endocrine disease), pre-admission comorbidities (ASCVD, diabetes, hypertension, heart failure, chronic obstructive pulmonary disease and renal dysfunction), medication (lipid-lowering therapy, antiplatelet drug and anticoagulant), mechanical ventilation and dialysis. ASCVD, atherosclerosis cardiovascular diseases. COPD, chronic obstructive pulmonary disease. OR, odds ratio. CI, confidence intervals.

eTable 5. Unadjusted and adjusted odds ratios for cardiovascular mortality by quintiles of LDL-C, HDL-C, total cholesterol and triglyceride levels

**Cardiovascular mortality**	**Quintile 1**	**Quintile 2**	**Quintile 3**	**Quintile 4**	**Quintile 5**
	OR (95% CI)	OR (95% CI)	OR (95% CI)	OR (95% CI)	OR (95% CI)
**LDL-C**					
Model I	1.91 (1.55, 2.34)	1.49 (1.20, 1.85)	1.24 (0.99, 1.55)	1.19 (0.95, 1.49)	reference
Model II	1.56 (1.26, 1.93)	1.24 (1.00, 1.54)	1.08 (0.86, 1.35)	1.08 (0.86, 1.35)	reference
Model III	1.17 (0.91, 1.50)	1.05 (0.82, 1.35)	0.96 (0.74, 1.23)	0.97 (0.76, 1.24)	reference
**HDL-C**					
Model I	1.11 (0.91, 1.36)	0.84 (0.68, 1.04)	0.89 (0.73, 1.10)	reference	1.06 (0.87, 1.29)
Model II	1.28 (1.05, 1.57)	0.93 (0.75, 1.15)	0.94 (0.77, 1.16)	reference	1.01 (0.83, 1.23)
Model III	1.24 (0.98, 1.57)	0.88 (0.69, 1.11)	0.87 (0.70, 1.10)	reference	0.96 (0.77, 1.20)
**Total cholesterol**					
Model I	1.64 (1.34, 2.01)	1.40 (1.14, 1.72)	1.20 (0.97, 1.49)	reference	0.95 (0.75, 1.18)
Model II	1.49 (1.21, 1.83)	1.28 (1.04, 1.57)	1.14 (0.92, 1.42)	reference	1.03 (0.82, 1.30)
Model III	1.38 (0.90, 1.79)	1.21 (0.95, 1.53)	1.07 (0.84, 1.36)	reference	1.09 (0.85, 1.40)
**Triglyceride**					
Model I	1.32 (1.08, 1.62)	1.26 (1.03, 1.55)	1.19 (0.97, 1.47)	reference	0.98 (0.79, 1.22)
Model II	1.13 (0.92, 1.39)	1.10 (0.89, 1.36)	1.10 (0.90, 1.36)	reference	1.10 (0.88, 1.37)
Model III	1.09 (0.78, 1.54)	1.05 (0.79, 1.41)	1.08 (0.84, 1.39)	reference	1.15 (0.82, 1.63)

Model I, unadjusted model; Model II, all cause mortality adjusted by age, sex and ethnicity; Model III, all cause mortality adjusted by age, sex, ethnicity, body mass index, heart rate, mean blood pressure, disease severity score (APACHE score and Glasgow coma score), lab measurements (LDL-C, HDL-C, total cholesterol, triglyceride, white blood cell, hemoglobin, platelet and albumin), primary disease of admission (circulatory disease, respiratory disease, digestive disease, neurological disease, genitourinary disease and endocrine disease), pre-admission comorbidities (ASCVD, diabetes, hypertension, heart failure, chronic obstructive pulmonary disease and renal dysfunction), medication (lipid-lowering therapy, antiplatelet drug and anticoagulant), mechanical ventilation and dialysis. ASCVD, atherosclerosis cardiovascular diseases. COPD, chronic obstructive pulmonary disease. OR, odds ratio. CI, confidence intervals.

eTable 6. Unadjusted and adjusted odds ratios for non-cardiovascular mortality by quintiles of LDL-C, HDL-C, total cholesterol and triglyceride levels

**Noncardiovascular mortality**	**Quintile 1**	**Quintile 2**	**Quintile 3**	**Quintile 4**	**Quintile 5**
	OR (95% CI)	OR (95% CI)	OR (95% CI)	OR (95% CI)	OR (95% CI)
**LDL-C**					
Model I	6.33 (4.72, 8.50)	3.20 (2.34, 4.36)	2.53 (1.84, 3.49)	1.65 (1.17, 2.33)	reference

Model II	5.73 (4.26, 7.71)	2.91 (2.13, 3.98)	2.36 (1.71, 3.26)	1.57 (1.12, 2.22)	reference
Model III	1.88 (1.36, 2.61)	1.57 (1.12, 2.18)	1.64 (1.17, 2.29)	1.27 (0.89, 1.82)	reference
**HDL-C**					
Model I	2.93 (2.33, 3.67)	1.35 (1.05, 1.75)	1.01 (0.77, 1.33)	reference	1.11 (0.85, 1.45)
Model II	3.34 (2.66, 4.21)	1.49 (1.15, 1.93)	1.07 (0.81, 1.40)	reference	1.05 (0.80, 1.37)
Model III	1.44 (1.10, 1.87)	1.23 (0.93, 1.64)	1.14 (0.85, 1.54)	reference	1.07 (0.80, 1.43)
**Total cholesterol**					
Model I	4.01 (3.16, 5.09)	1.92 (1.48, 2.49)	1.30 (0.98, 1.72)	reference	0.65 (0.47, 0.91)
Model II	3.88 (3.05, 4.93)	1.85 (1.43, 2.40)	1.26 (0.95, 1.67)	reference	0.68 (0.48, 0.95)
Model III	1.59 (1.17, 2.16)	1.36 (1.01, 1.83)	1.15 (0.84, 1.57)	reference	0.79 (0.55, 1.15)
**Triglyceride**					
Model I	1.05 (0.84, 1.33)	1.11 (0.89, 1.40)	1.06 (0.85, 1.34)	reference	0.91 (0.72, 1.16)
Model II	0.95 (0.75, 1.20)	1.02 (0.81, 1.28)	1.01 (0.80, 1.27)	reference	0.98 (0.77, 1.25)
Model III	0.94 (0.68, 1.29)	1.06 (0.79, 1.41)	1.12 (0.86, 1.47)	reference	0.93 (0.67, 1.28)

Model I, unadjusted model; Model II, all cause mortality adjusted by age, sex and ethnicity; Model III, all cause mortality adjusted by age, sex, ethnicity, body mass index, heart rate, mean blood pressure, disease severity score (APACHE score and Glasgow coma score), lab measurements (LDL-C, HDL-C, total cholesterol, triglyceride, white blood cell, hemoglobin, platelet and albumin), primary disease of admission (circulatory disease, respiratory disease, digestive disease, neurological disease, genitourinary disease and endocrine disease), pre-admission comorbidities (ASCVD, diabetes, hypertension, heart failure, chronic obstructive pulmonary disease and renal dysfunction), medication (lipid-lowering therapy, antiplatelet drug and anticoagulant), mechanical ventilation and dialysis. ASCVD, atherosclerosis cardiovascular diseases. COPD, chronic obstructive pulmonary disease. OR, odds ratio. CI, confidence intervals.

eTable 7. Univariable and multivariable analysis for all-cause mortality with lipid levels below or above the risk inflection point

Lipid level	Model I<br>OR (95% CI)	Model II<br>OR (95% CI)	Model III<br>OR (95% CI)
**LDL-C level**			
Increase per 10 mg/dL (≤96 mg/dL)	0.84 (0.81, 0.86)	0.85 (0.81, 0.87)	0.92 (0.89, 0.95)
Increase per 10 mg/dL (>96 mg/dL)	0.97 (0.92, 1.01)	0.99 (0.97, 1.03)	1.03 (0.97, 1.06)
**HDL-C level**			
Increase per 10 mg/dL (≤27 mg/dL)	0.52 (0.45, 0.60)	0.51 (0.41, 0.60)	0.64 (0.50, 0.75)
Increase per 10 mg/dL (>27 mg/dL)	1.04 (1.00, 1.09)	1.01 (0.95, 1.05)	1.02 (0.95, 1.06)
**total cholesterol level**			
Increase per 10 mg/dL (≤140 mg/dL)	0.85 (0.80, 0.87)	0.85 (0.82, 0.87)	0.91 (0.86, 0.96)
Increase per 10 mg/dL (>140 mg/dL)	0.95(0.94, 0.98)	0.98 (0.93, 1.01)	0.99 (0.97, 1.03)

Model I, unadjusted model; Model II, all cause mortality adjusted by age, sex and ethnicity; Model III, all cause mortality adjusted by age, sex, ethnicity, body mass index, heart rate, mean blood pressure, disease severity score (APACHE score and Glasgow coma score), lab measurements (LDL-C, HDL-C, total cholesterol, triglyceride, white blood cell, hemoglobin, platelet and albumin), primary disease of admission (circulatory disease, respiratory disease, digestive disease, neurological disease, genitourinary disease and endocrine disease), pre-admission comorbidities (ASCVD, diabetes, hypertension, heart failure, chronic obstructive pulmonary disease and renal

dysfunction), medication (lipid-lowering therapy, antiplatelet drug and anticoagulant), mechanical ventilation and dialysis. ASCVD, atherosclerosis cardiovascular diseases. COPD, chronic obstructive pulmonary disease. OR, odds ratio. CI, confidence intervals.

eTable 8. Unadjusted and adjusted odds ratios for all-cause mortality by LDL-C level combined with HDL-C level

Lipid level	Model I<br>OR (95% CI)	Model II<br>OR (95% CI)	Model III<br>OR (95% CI)
**All-cause mortality**			
LDL≤96, HDL≤27 mg/dL	3.03 (2.60, 3.54)	3.04 (2.60, 3.54)	1.52 (1.26, 1.82)
LDL>96, HDL≤27 mg/dL	1.75 (1.53, 1.99)	1.53 (1.34, 1.75)	1.43 (1.02, 1.92)
LDL≤96, HDL>27 mg/dL	1.50 (1.09, 2.08)	1.79 (1.29, 2.49)	1.22 (1.06, 1.41)
LDL>96, HDL>27 mg/dL	reference	reference	reference
**Cardiovascular mortality**			
LDL≤96, HDL≤27 mg/dL	1.61 (1.32, 1.98)	1.59 (1.29, 1.95)	1.37 (1.07, 1.76)
LDL>96, HDL≤27 mg/dL	1.35 (1.15, 1.57)	1.16 (0.99, 1.35)	1.00 (0.59, 1.68)
LDL≤96, HDL>27 mg/dL	0.91 (0.57, 1.45)	1.10 (0.69, 1.75)	1.02 (0.86, 1.22)
LDL>96, HDL>27 mg/dL	reference	reference	reference
**Noncardiovascular mortality**			
LDL≤96, HDL≤27 mg/dL	6.55 (5.09, 8.43)	6.58 (5.11, 8.48)	1.82 (1.37, 2.43)
LDL>96, HDL≤27 mg/dL	2.75 (2.17, 3.48)	2.48 (1.96, 3.15)	1.93 (1.18, 2.88)
LDL≤96, HDL>27 mg/dL	3.08 (1.94, 4.87)	3.56 (2.24, 5.64)	1.64 (1.27, 2.10)
LDL>96, HDL>27 mg/dL	reference	reference	reference

Model I, unadjusted model; Model II, all cause mortality adjusted by age, sex and ethnicity; Model III, all cause mortality adjusted by age, sex, ethnicity, body mass index, heart rate, mean blood pressure, disease severity score (APACHE score and Glasgow coma score), lab measurements (LDL-C, HDL-C, total cholesterol, triglyceride, white blood cell, hemoglobin, platelet and albumin), primary disease of admission (circulatory disease, respiratory disease, digestive disease, neurological disease, genitourinary disease and endocrine disease), pre-admission comorbidities (ASCVD, diabetes, hypertension, heart failure, chronic obstructive pulmonary disease and renal dysfunction), medication (lipid-lowering therapy, antiplatelet drug and anticoagulant), mechanical ventilation and dialysis. ASCVD, atherosclerosis cardiovascular diseases. COPD, chronic obstructive pulmonary disease. OR, odds ratio. CI, confidence intervals.

eTable 9. Unadjusted and adjusted odds ratios for all-cause mortality according to quintiles of LDL-C, HDL-C, total cholesterol and triglyceride levels by excluding death within the first 24 hours of ICU entry (n=27097)

**All-cause mortality**	**Quintile 1**<br>OR (95% CI)	**Quintile 2**<br>OR (95% CI)	**Quintile 3**<br>OR (95% CI)	**Quintile 4**<br>OR (95% CI)	**Quintile 5**<br>OR (95% CI)
**LDL-C**					
Model I	3.33 (2.78, 3.98)	2.07 (1.71, 2.50)	1.69 (1.39, 2.06)	1.32 (1.08, 1.62)	reference
Model II	2.88 (2.40, 3.46)	1.81 (1.49, 2.19)	1.53 (1.26, 1.86)	1.23 (1.00, 1.51)	reference
Model III	1.57 (1.28, 1.93)	1.31 (1.06, 1.61)	1.25 (1.01, 1.54)	1.09 (0.88, 1.35)	reference

**HDL-C**					
Model I	1.82 (1.55, 2.13)	1.07 (0.90, 1.27)	0.96 (0.81, 1.15)	reference	1.11 (0.94, 1.32)
Model II	2.09 (1.77, 2.45)	1.17 (0.98, 1.40)	1.01 (0.85, 1.21)	reference	1.06 (0.89, 1.25)
Model III	1.25 (1.05, 1.44)	1.00 (0.83, 1.21)	0.94 (0.78, 1.13)	reference	1.05 (0.87, 1.26)
**Total cholesterol**					
Model I	2.52 (2.14, 2.97)	1.62 (1.36, 1.92)	1.25 (1.04, 1.50)	reference	0.79 (0.65, 0.97)
Model II	2.37 (2.01, 2.79)	1.52 (1.28, 1.81)	1.20 (1.00, 1.44)	reference	0.84 (0.69, 1.03)
Model III	1.42 (1.18, 1.71)	1.24 (1.03, 1.50)	1.10 (0.91, 1.34)	reference	0.90 (0.73, 1.12)
**Triglyceride**					
Model I	1.18 (1.00, 1.40)	1.16 (0.98, 1.37)	1.15 (0.98, 1.36)	reference	0.95 (0.80, 1.13)
Model II	1.04 (0.88, 1.22)	1.03 (0.87, 1.22)	1.08 (0.91, 1.27)	reference	1.04 (0.87, 1.24)
Model III	1.11 (0.92, 1.33)	1.08 (0.90, 1.30)	1.11 (0.93, 1.33)	reference	0.98 (0.81, 1.19)

Model I, unadjusted model; Model II, all cause mortality adjusted by age, sex and ethnicity; Model III, all cause mortality adjusted by age, sex, ethnicity, body mass index, heart rate, mean blood pressure, disease severity score (APACHE score and Glasgow coma score), lab measurements (LDL-C, HDL-C, total cholesterol, triglyceride, white blood cell, hemoglobin, platelet and albumin), primary disease of admission (circulatory disease, respiratory disease, digestive disease, neurological disease, genitourinary disease and endocrine disease), pre-admission comorbidities (ASCVD, diabetes, hypertension, heart failure, chronic obstructive pulmonary disease and renal dysfunction), medication (lipid-lowering therapy, antiplatelet drug and anticoagulant), mechanical ventilation and dialysis. ASCVD, atherosclerosis cardiovascular diseases. COPD, chronic obstructive pulmonary disease. OR, odds ratio. CI, confidence intervals.

eTable 10. Unadjusted and adjusted odds ratios for all-cause mortality according to quintiles of LDL-C, HDL-C, total cholesterol and triglyceride levels with complete-case analyse (n=21223)

All-cause mortality	Quintile 1	Quintile 2	Quintile 3	Quintile 4	Quintile 5
	OR (95% CI)	OR (95% CI)	OR (95% CI)	OR (95% CI)	OR (95% CI)
**LDL-C**					
Model I	2.95 (2.44, 3.58)	1.87 (1.53, 2.29)	1.46 (1.18, 1.80)	1.21 (0.97, 1.50)	reference
Model II	2.51 (2.06, 3.05)	1.61 (1.31, 1.97)	1.29 (1.04, 1.59)	1.12 (0.90, 1.39)	reference
Model III	1.29 (1.07, 1.58)	1.16 (0.93, 1.46)	1.10 (0.87, 1.38)	0.99 (0.78, 1.26)	reference
**HDL-C**					
Model I	1.66 (1.40, 1.98)	1.01 (0.84, 1.22)	0.88 (0.72, 1.07)	1.11 (0.92, 1.34)	1.08 (0.90, 1.30)
Model II	1.89 (1.58, 2.25)	1.10 (0.91, 1.34)	0.92 (0.76, 1.12)	1.05 (0.87, 1.27)	1.03 (0.85, 1.24)
Model III	1.28 (1.10, 1.48)	0.92 (0.75, 1.14)	0.86 (0.70, 1.06)	1.11 (0.91, 1.37)	1.00 (0.81, 1.22)
**Total cholesterol**					
Model I	2.55 (2.13, 3.05)	1.60 (1.32, 1.94)	1.16 (0.94, 1.42)	reference	0.90 (0.72, 1.12)
Model II	2.37 (1.97, 2.84)	1.49 (1.23, 1.80)	1.10 (0.90, 1.36)	reference	0.97 (0.78, 1.20)
Model III	1.31 (1.04, 1.66)	1.18 (0.95, 1.47)	0.99 (0.79, 1.24)	reference	0.98 (0.77, 1.25)
**Triglyceride**					
Model I	1.38 (1.15, 1.66)	1.31 (1.09, 1.58)	1.32 (1.09, 1.59)	reference	1.03 (0.85, 1.26)
Model II	1.21 (1.00, 1.46)	1.17 (0.97, 1.41)	1.23 (1.02, 1.49)	reference	1.15 (0.94, 1.40)
Model III	1.29 (0.99, 1.67)	1.23 (0.97, 1.55)	1.28 (0.89, 1.59)	reference	1.07 (0.81, 1.40)

Model I, unadjusted model; Model II, all cause mortality adjusted by age, sex and ethnicity; Model III, all cause

mortality adjusted by age, sex, ethnicity, body mass index, heart rate, mean blood pressure, disease severity score (APACHE score and Glasgow coma score), lab measurements (LDL-C, HDL-C, total cholesterol, triglyceride, white blood cell, hemoglobin, platelet and albumin), primary disease of admission (circulatory disease, respiratory disease, digestive disease, neurological disease, genitourinary disease and endocrine disease), pre-admission comorbidities (ASCVD, diabetes, hypertension, heart failure, chronic obstructive pulmonary disease and renal dysfunction), medication (lipid-lowering therapy, antiplatelet drug and anticoagulant), mechanical ventilation and dialysis. ASCVD, atherosclerosis cardiovascular diseases. COPD, chronic obstructive pulmonary disease. OR, odds ratio. CI, confidence intervals.

eTable 11. Unadjusted and adjusted odds ratios for all-cause mortality according to quintiles of LDL-C, HDL-C, total cholesterol and triglyceride levels by using the first measurement of ICU admission (n=20388)

All-cause mortality	Quintile 1	Quintile 2	Quintile 3	Quintile 4	Quintile 5
	OR (95% CI)	OR (95% CI)	OR (95% CI)	OR (95% CI)	OR (95% CI)
**LDL-C**					
Model I	3.25 (2.68, 3.94)	2.05 (1.67, 2.51)	1.67 (1.36, 2.07)	1.32 (1.06, 1.64)	reference
Model II	2.82 (2.32, 3.43)	1.80 (1.47, 2.21)	1.52 (1.23, 1.88)	1.23 (0.99, 1.54)	reference
Model III	1.77 (1.44, 2.18)	1.39 (1.12, 1.72)	1.29 (1.03, 1.60)	1.11 (0.88, 1.39)	reference
**HDL-C**					
Model I	1.95 (1.64, 2.33)	1.12 (0.92, 1.35)	1.04 (0.86, 1.26)	reference	1.15 (0.95, 1.39)
Model II	2.22 (1.86, 2.65)	1.22 (1.01, 1.48)	1.09 (0.90, 1.32)	reference	1.10 (0.91, 1.32)
Model III	1.53 (1.26, 1.85)	1.09 (0.89, 1.33)	1.03 (0.84, 1.26)	reference	1.05 (0.86, 1.28)
**Total cholesterol**					
Model I	2.52 (2.11, 3.00)	1.59 (1.32, 1.91)	1.24 (1.02, 1.51)	reference	0.78 (0.62, 0.97)
Model II	2.36 (1.98, 2.82)	1.49 (1.24, 1.80)	1.19 (0.98, 1.45)	reference	0.82 (0.66, 1.03)
Model III	1.63 (1.35, 1.98)	1.28 (1.05, 1.56)	1.13 (0.92, 1.38)	reference	0.88 (0.70, 1.11)
**Triglyceride**					
Model I	1.19 (1.00, 1.42)	1.23 (1.03, 1.47)	1.15 (0.96, 1.38)	reference	0.93 (0.77, 1.12)
Model II	1.05 (0.88, 1.26)	1.10 (0.92, 1.32)	1.08 (0.91, 1.30)	reference	1.01 (0.84, 1.22)
Model III	1.09 (0.90, 1.32)	1.13 (0.94, 1.37)	1.11 (0.92, 1.35)	reference	0.96 (0.79, 1.18)

Model I, unadjusted model; Model II, all cause mortality adjusted by age, sex and ethnicity; Model III, all cause mortality adjusted by age, sex, ethnicity, body mass index, heart rate, mean blood pressure, disease severity score (APACHE score and Glasgow coma score), lab measurements (LDL-C, HDL-C, total cholesterol, triglyceride, white blood cell, hemoglobin, platelet and albumin), primary disease of admission (circulatory disease, respiratory disease, digestive disease, neurological disease, genitourinary disease and endocrine disease), pre-admission comorbidities (ASCVD, diabetes, hypertension, heart failure, chronic obstructive pulmonary disease and renal dysfunction), medication (lipid-lowering therapy, antiplatelet drug and anticoagulant), mechanical ventilation and dialysis. ASCVD, atherosclerosis cardiovascular diseases. COPD, chronic obstructive pulmonary disease. OR, odds ratio. CI, confidence intervals.

eFigure 1. Multivariable adjusted odds ratios for all-cause and cause-specific mortality by quintiles of LDL-C, HDL-C, total cholesterol and triglyceride levels in individuals with lipid-lowering therapy

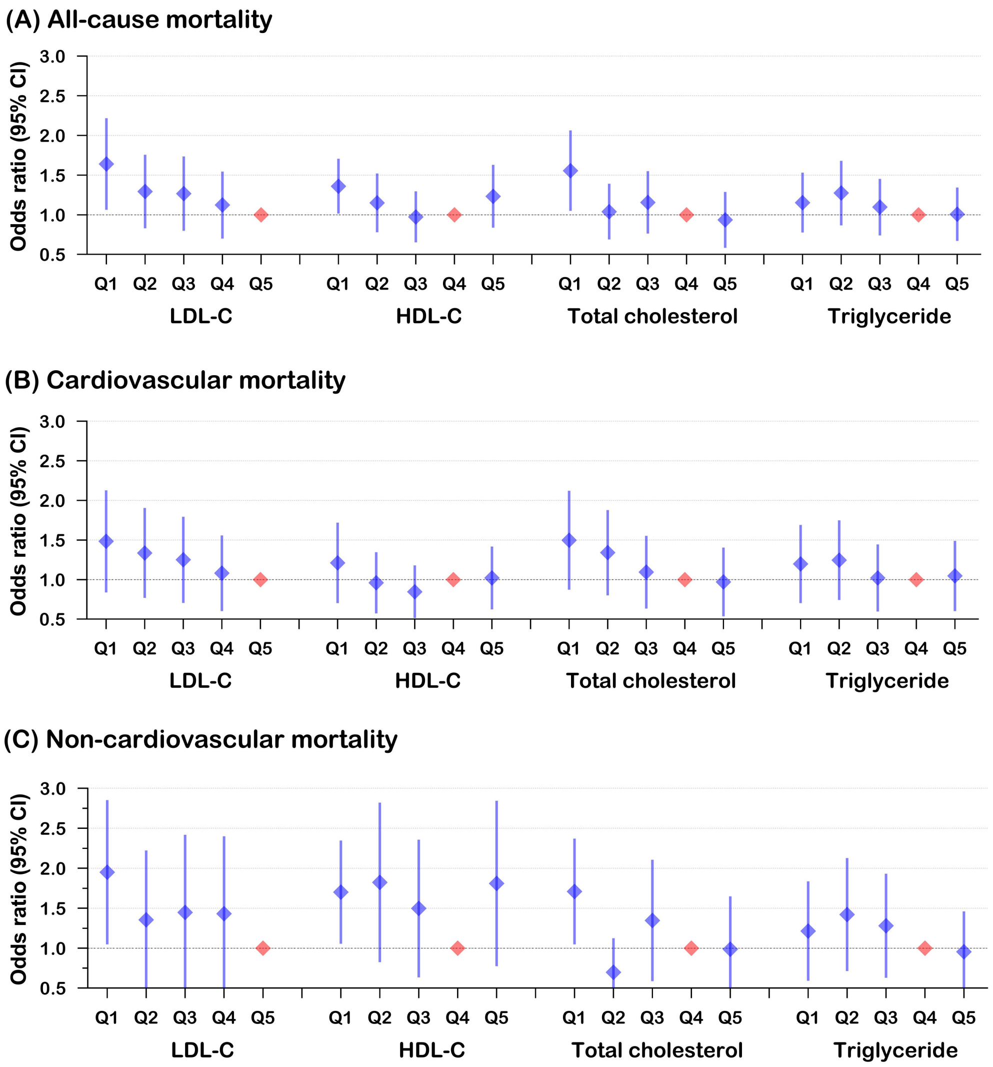

All-cause mortality (A), cardiovascular mortality (B) and non-cardiovascular mortality (C) adjusted by age, sex, ethnicity, body mass index, heart rate, mean blood pressure, disease severity score (APACHE score and Glasgow coma score), lab measurements (LDL-C, HDL-C, total cholesterol, triglyceride, white blood cell, hemoglobin, platelet and albumin), primary disease of admission (circulatory disease, respiratory disease, digestive disease, neurological disease, genitourinary disease and endocrine disease), pre-admission comorbidities (ASCVD, diabetes, hypertension, heart failure, chronic obstructive pulmonary disease and renal dysfunction), medication (lipid-lowering therapy, antiplatelet drug and anticoagulant), mechanical ventilation and dialysis. ASCVD, atherosclerosis cardiovascular diseases. COPD, chronic obstructive pulmonary disease.

eFigure 2. Probability of all cause and cause-specific mortality based on LDL-C, HDL-C, total cholesterol and triglyceride levels in individuals without lipid-lowering therapy

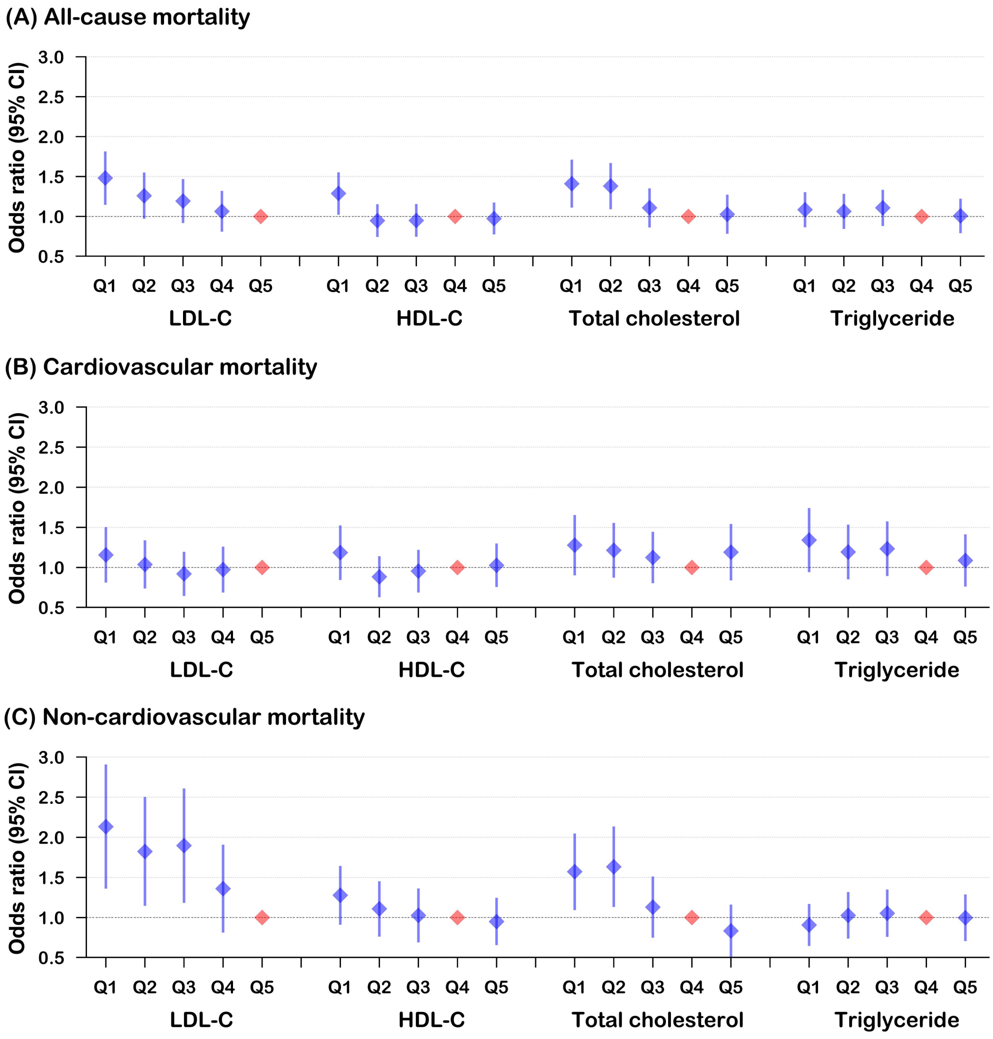

All-cause mortality (A), cardiovascular mortality (B) and non-cardiovascular mortality (C) adjusted by age, sex, ethnicity, body mass index, heart rate, mean blood pressure, disease severity score (APACHE score and Glasgow coma score), lab measurements (LDL-C, HDL-C, total cholesterol, triglyceride, white blood cell, hemoglobin, platelet and albumin), primary disease of admission (circulatory disease, respiratory disease, digestive disease, neurological disease, genitourinary disease and endocrine disease), pre-admission comorbidities (ASCVD, diabetes, hypertension, heart failure, chronic obstructive pulmonary disease and renal dysfunction), medication (lipid-lowering therapy, antiplatelet drug and anticoagulant), mechanical ventilation and dialysis. ASCVD, atherosclerosis cardiovascular diseases. COPD, chronic obstructive pulmonary disease.

eFigure 3. Multivariable adjusted all-cause mortality risk for total cholesterol levels stratified by covariates

Forest Plot of odds ratio for all-cause mortality stratified by age, sex, ethnicity, lipid-lowering therapy, ASCVD, LDL-C and HDL-C levels, with the first quintile (≤110 mg/dL) compared to the fourth quintile of TC levels (159-188 mg/dL). The model was adjusted byage, sex, ethnicity, body mass index, heart rate, mean blood pressure, disease severity score (APACHE score and Glasgow coma score), lab measurements (LDL-C, HDL-C, total cholesterol, triglyceride, white blood cell, hemoglobin, platelet and albumin), primary disease of admission (circulatory disease, respiratory disease, digestive disease, neurological disease, genitourinary disease and endocrine disease), pre-admission comorbidities (ASCVD, diabetes, hypertension, heart failure, chronic obstructive pulmonary disease and renal dysfunction), medication (lipid-lowering therapy, antiplatelet drug and anticoagulant), mechanical ventilation and dialysis. ASCVD, atherosclerosis cardiovascular diseases. COPD, chronic obstructive pulmonary disease.

Variable	OR (95% CI)	P for interaction
**Age**		
>65 years	1.20 (1.03- 1.40)	0.14
≤65 years	1.38 (1.10- 1.73)	
**Sex**		
Male	1.27 (1.07- 1.52)	0.30
Female	1.26 (1.04- 1.53)	
**Ethnicity**		
Caucasian	1.26 (1.09- 1.46)	0.76
Others	1.34 (1.02- 1.75)	
**Lipid-lowering**		
Yes	1.18 (0.93- 1.50)	0.64
No	1.31 (1.12- 1.53)	
**ASCVD**		
Yes	1.27 (1.02- 1.59)	0.53
No	1.29 (1.10- 1.51)	
**HDL-C**		
≤27 mg/dL	1.44 (1.04- 1.99)	0.22
>27 mg/dL	1.21 (1.06- 1.38)	

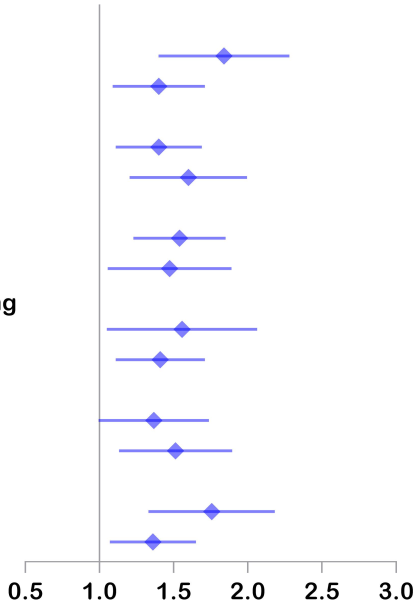

eFigure 4. Probability of all cause mortality based on LDL-C, HDL-C, total cholesterol and triglyceride levels by excluding death within the first 24 hours of ICU entry

Sensitivity analysis by excluding death within the first 24 hours of ICU entry (n=27097). All-cause mortality adjusted by age, sex, ethnicity, body mass index, heart rate, mean blood pressure, disease severity score (APACHE score and Glasgow coma score), lab measurements (LDL-C, HDL-C, total cholesterol, triglyceride, white blood cell, hemoglobin, platelet and albumin), primary disease of admission (circulatory disease, respiratory disease, digestive disease, neurological disease, genitourinary disease and endocrine disease), pre-admission comorbidities (ASCVD, diabetes, hypertension, heart failure, chronic obstructive pulmonary disease and renal dysfunction), medication (lipid-lowering therapy, antiplatelet drug and anticoagulant), mechanical ventilation and dialysis. ASCVD, atherosclerosis cardiovascular diseases. COPD, chronic obstructive pulmonary disease.

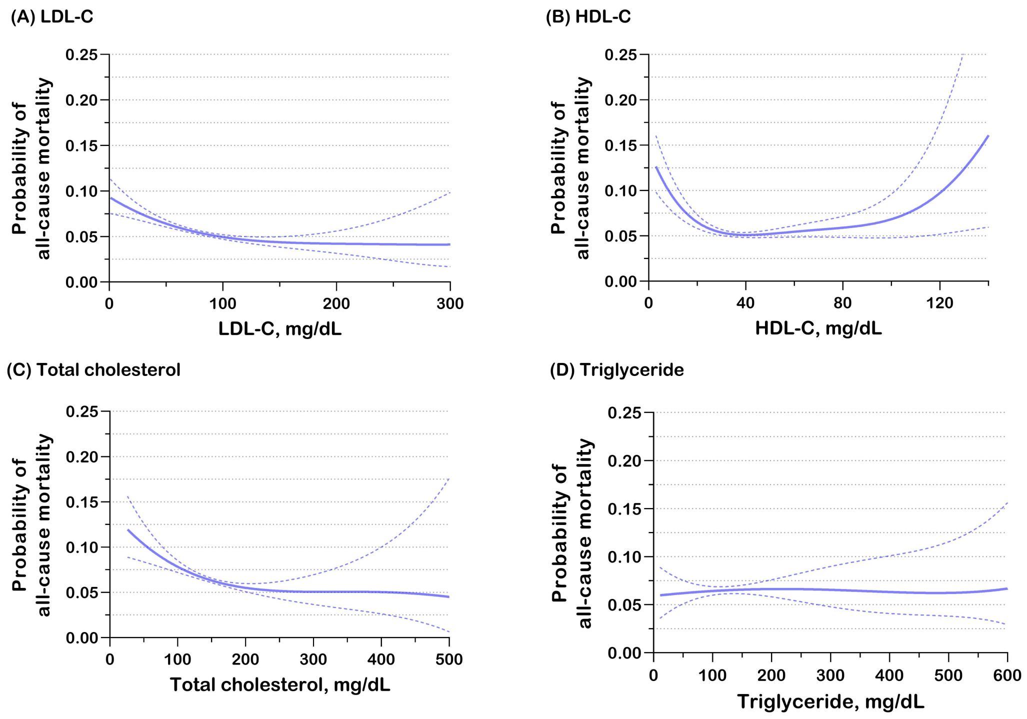

eFigure 5. Probability of all cause mortality based on LDL-C, HDL-C, total cholesterol and triglyceride levels with complete-case analyse

Sensitivity analysis by excluding cases with missing data (n=21223). All-cause mortality adjusted by age, sex, ethnicity, body mass index, heart rate, mean blood pressure, disease severity score (APACHE score and Glasgow coma score), lab measurements (LDL-C, HDL-C, total cholesterol, triglyceride, white blood cell, hemoglobin, platelet and albumin), primary disease of admission (circulatory disease, respiratory disease, digestive disease, neurological disease, genitourinary disease and endocrine disease), pre-admission comorbidities (ASCVD, diabetes, hypertension, heart failure, chronic obstructive pulmonary disease and renal dysfunction), medication (lipid-lowering therapy, antiplatelet drug and anticoagulant), mechanical ventilation and dialysis. ASCVD, atherosclerosis cardiovascular diseases. COPD, chronic obstructive pulmonary disease.

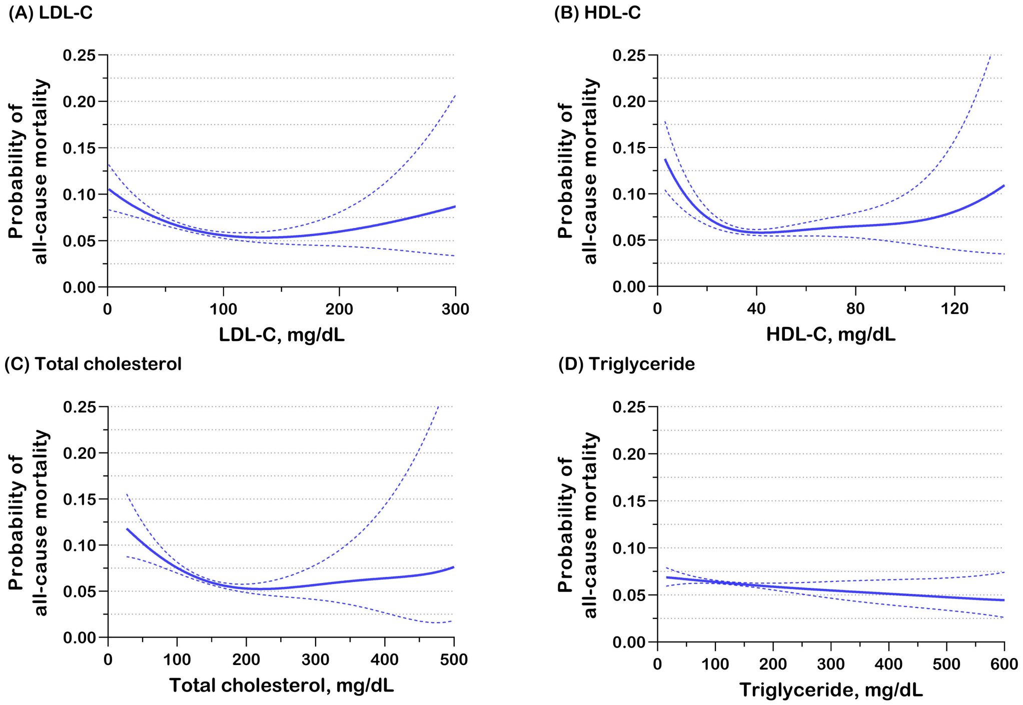

eFigure 6. Probability of all cause mortality based on LDL-C, HDL-C, total cholesterol and triglyceride levels by using the first measurement of ICU admission

Sensitivity analysis by using the first measurement of lipid levels after ICU admission instead of the first measurement of hospital admission (n=20388). All-cause mortality adjusted by age, sex, ethnicity, body mass index, heart rate, mean blood pressure, disease severity score (APACHE score and Glasgow coma score), lab measurements (LDL-C, HDL-C, total cholesterol, triglyceride, white blood cell, hemoglobin, platelet and albumin), primary disease of admission (circulatory disease, respiratory disease, digestive disease, neurological disease, genitourinary disease and endocrine disease), pre-admission comorbidities (ASCVD, diabetes, hypertension, heart failure, chronic obstructive pulmonary disease and renal dysfunction), medication (lipid-lowering therapy, antiplatelet drug and anticoagulant), mechanical ventilation and dialysis. ASCVD, atherosclerosis cardiovascular diseases. COPD, chronic obstructive pulmonary disease.

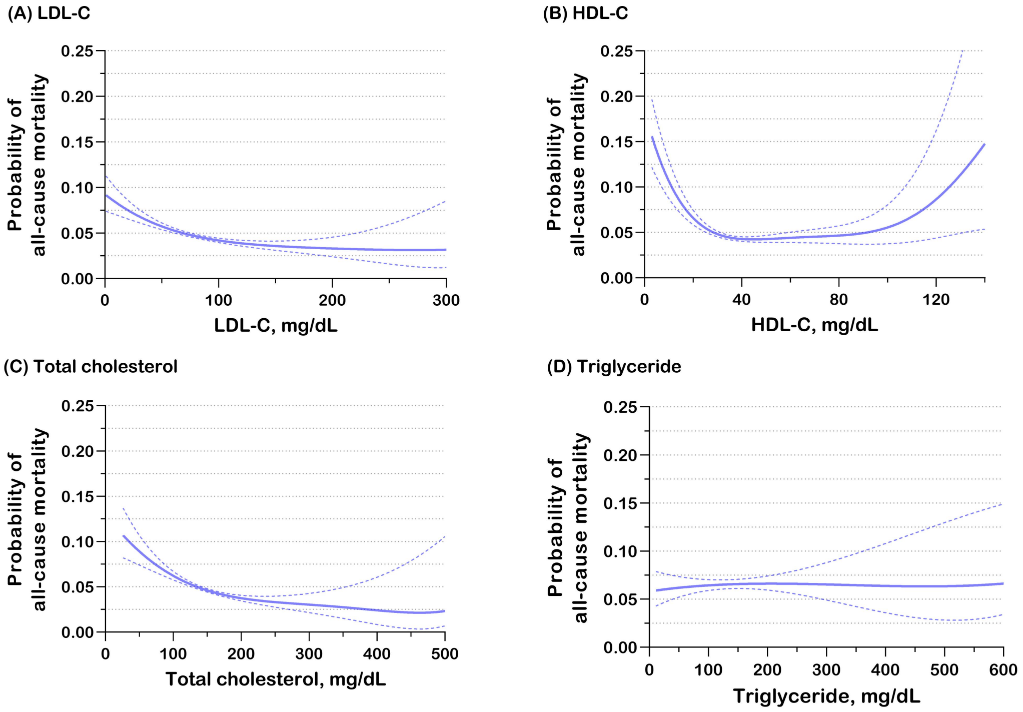

eFigure 7. Kaplan-Meier curve for cumulative incidence with all cause mortality by quintiles of lipid levels

(A)

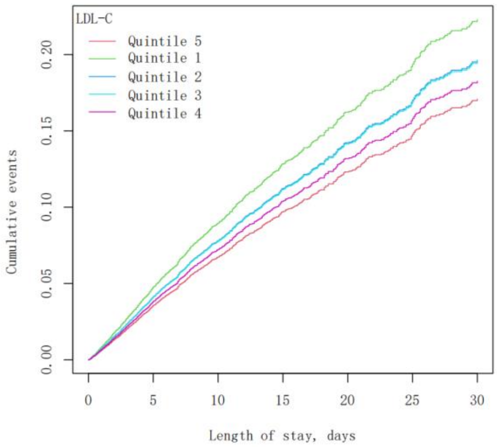

(B)

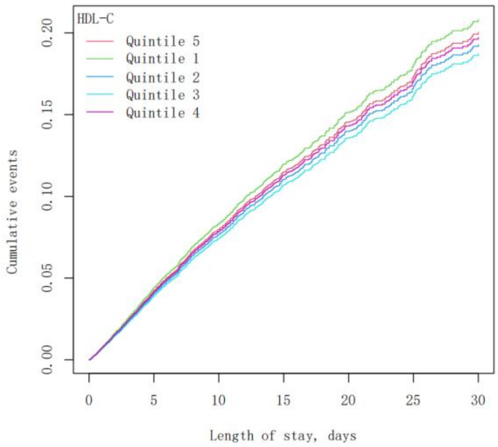

(C)

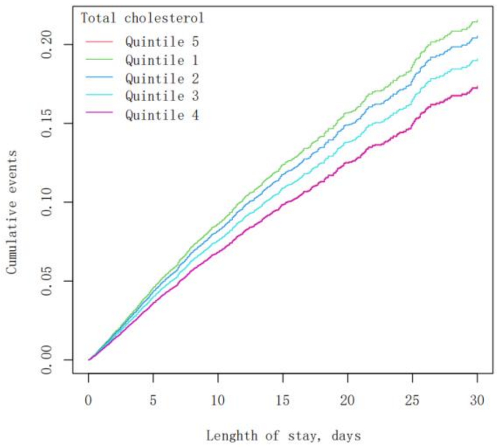

(D)

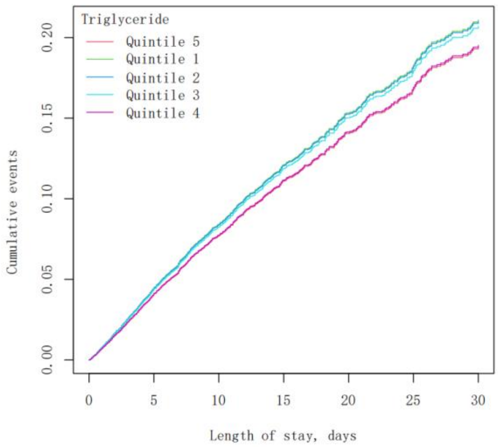

eFigure 8. Kaplan–Meier curve for cumulative incidence with all cause mortality by LDL-C level combined with HDL-C level

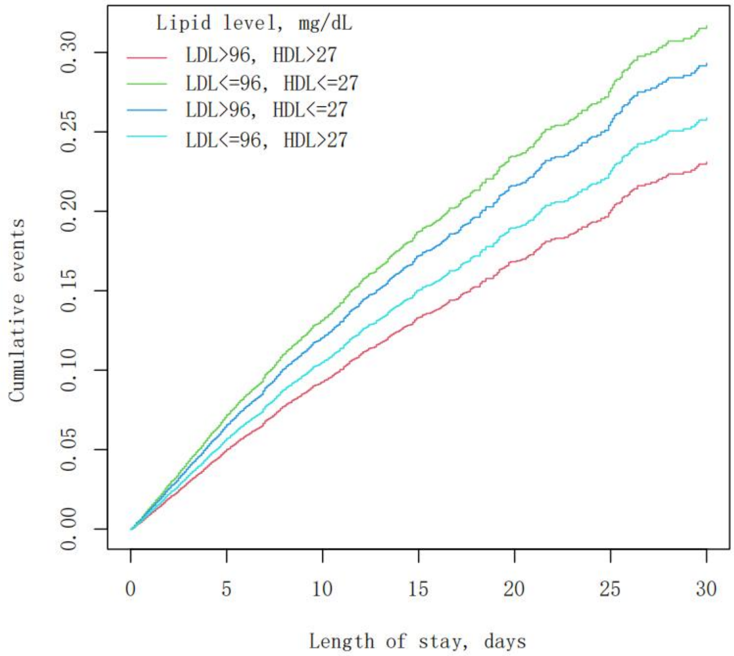

Supplement: Supplementary file 1 — Supplementary Information. [file 41598_2023_32209_MOESM1_ESM.pdf]
